# The Role of Long Noncoding RNAs on Male Infertility: A Systematic Review and In Silico Analysis

**DOI:** 10.3390/biology11101510

**Published:** 2022-10-15

**Authors:** Maria-Anna Kyrgiafini, Theologia Sarafidou, Zissis Mamuris

**Affiliations:** Laboratory of Genetics, Comparative and Evolutionary Biology, Department of Biochemistry and Biotechnology, University of Thessaly, Viopolis, Mezourlo, 41500 Larissa, Greece

**Keywords:** male infertility, long noncoding RNAs (lncRNAs), in silico, asthenozoospermia, teratozoospermia, oligozoospermia, azoospermia, cancer

## Abstract

**Simple Summary:**

Male infertility is a health problem affecting a huge number of couples worldwide. For many years, scientists have focused on protein-coding genes to explore the causes and mechanisms of male infertility. In addition, noncoding regions that are transcribed but do not lead to protein production have also been proven to play an important role in many cellular processes and diseases. The exact role of long noncoding RNAs on male infertility is not well understood. In order to address this gap, we performed a systematic review of the literature using two databases, and then a bioinformatics analysis to identify the most important long noncoding RNAs in male infertility, as well as their mechanism of action. At first, we identified 625 articles, but after selecting some of them according to the criteria that we defined, our final sample comprised 20 articles. After analyzing them, many long noncoding RNAs were found to be deregulated in male infertility or specific subtypes of it, paving the way for a better understanding of the molecular mechanisms behind male infertility, or even for the improvement of diagnosis and therapy. Finally, an association was found between male infertility and cancer that requires further investigation in the future.

**Abstract:**

Male infertility is a complex disorder affecting many couples worldwide. Long noncoding RNAs (lncRNAs) regulate important cellular processes; however, a comprehensive understanding of their role in male infertility is limited. This systematic review investigates the differential expressions of lncRNAs in male infertility or variations in lncRNA regions associated with it. The PRISMA guidelines were used to search Pubmed and Web of Science (1 June 2022). Inclusion criteria were human participants, patients diagnosed with male infertility, and English language speakers. We also performed an in silico analysis investigating lncRNAs that are reported in many subtypes of male infertility. A total of 625 articles were found, and after the screening and eligibility stages, 20 studies were included in the final sample. Many lncRNAs are deregulated in male infertility, and interactions between lncRNAs and miRNAs play an important role. However, there is a knowledge gap regarding the impact of variants found in lncRNA regions. Furthermore, eight lncRNAs were identified as differentially expressed in many subtypes of male infertility. After in silico analysis, gene ontology (GO) and KEGG enrichment analysis of the genes targeted by them revealed their association with bladder and prostate cancer. However, pathways involved in general in tumorigenesis and cancer development of all types, such as p53 pathways, apoptosis, and cell death, were also enriched, indicating a link between cancer and male infertility. This evidence, however, is preliminary. Future research is needed to explore the exact mechanism of action of the identified lncRNAs and investigate the association between male infertility and cancer.

## 1. Introduction

Infertility is considered a global health problem, as more than 186 million people are affected worldwide [1], and has serious personal, social, and economic consequences for the affected couples [2]. It is defined by the World Health Organization (WHO) as the inability to conceive after at least 12 months of regular, unprotected sexual intercourse. Though it is estimated that male factors are responsible for 20–30% of cases and contribute even to 50% [3], male infertility remains understudied in comparison with female fertility. It is a complex disorder affected by both genetic and environmental factors, and it can be classified into subcategories according to the sperm parameters that are affected. The World Health Organization (WHO) has defined some semen analysis reference values for classifying and precisely defining the main subtypes of male infertility. According to 2010 guidelines, the sperm reference values include a semen volume of 1.5 mL, sperm concentration of 15 million/mL, sperm total motility of 40%, sperm progressive motility of 32%, and sperm with a normal morphology of 4% (Kruger criteria). Therefore, asthenozoospermia is defined as a reduction in the total motility lower than the reference value (sperm total motility < 40% and sperm progressive motility < 32%), while oligozoospermia is observed when the sperm concentration is lower than 15 million/mL. Similarly, a sample is characterized as teratozoospermic when less than 4% of spermatozoa have normal morphology. More than one semen parameter can also be affected, leading to male infertility subtypes such as oligoasthenozoospermia (sperm concentration < 15 million/mL, sperm total motility < 40%, and sperm progressive motility < 32%) and teratoasthenozoospermia (normal morphology < 4%, sperm total motility < 40% and sperm progressive motility < 32%). Semen analysis (seminogram) remains the foundation of the male fertility evaluation and diagnosis which classifies men into the above sub-categories [3,4].

Although many genes have been associated with specific subtypes of male infertility [5], a large number of cases are characterized as idiopathic, and no cause can be identified [3]. Furthermore, genomes are extensively transcribed (>85%), but only a small percentage of these transcribed RNAs are translated into proteins [6]. For many years, these intergenic regions were considered “junk DNA,” but recently, it was discovered that all of these noncoding RNAs (ncRNAs) can have a functional role [7]. More specifically, ncRNAs can be classified into several categories, such as microRNAs (miRNAs), circular RNAs (circRNAs), long noncoding RNAs (lncRNAs), PIWI-interacting RNAs (piRNAs), etc. LncRNAs are transcripts longer than 200 nucleotides that are located in the cytoplasm or the nucleus and affect gene expression through transcriptional, post-transcriptional, and epigenetic regulation [8,9]. Furthermore, they exert their regulatory role through interactions with proteins and transcriptional factors, mRNAs, and miRNAs, and it has been observed that they have tissue-specific and condition-specific expression patterns [10].

In recent years, the interest in lncRNAs has increased rapidly, and the underlying mechanisms that regulate reproduction through lncRNAs are poorly understood or are under debate [11]. Μany platforms have been developed for the detection and measurement of noncoding RNAs, such as real-time quantitative PCR (qPCR), microarray-based methods, and, more recently, high-throughput next-generation sequencing (NGS). Growing evidence also suggests their involvement in the pathogenesis of several complex diseases, such as diabetes mellitus [12], cardiovascular diseases [13], neurodegenerative diseases [14], etc. Regarding reproduction, lncRNAs are involved in the differentiation, proliferation, and self-renewal of spermatogonial stem cells [15], and although more and more studies explore their role in reproduction both in human subjects and model animals, it is not clear exactly how they contribute to male infertility.

Therefore, our understanding of the role of lncRNAs in male infertility is limited. There are many heterogeneous studies reporting findings on lncRNAs and fertility, but to our knowledge, there are no systematic reviews summarizing the lncRNAs that have been associated with male infertility, or that are deregulated in specific subtypes and thus potentially involved in the pathogenesis process. A comparison of the studies and their findings could be important to identify similar patterns of expression and the lncRNAs that play an important role in male infertility.

In order to address this gap and provide a comprehensive view of the available evidence, we undertake what is, to our knowledge, a systematic review of studies conducted only on human subjects, due to limited evolutionary conservation of lncRNAs between species [16], that investigates the role of lncRNAs in male infertility. In the present systematic review, we include articles which studied the differential expression of lncRNAs in male infertility, as well as studies reporting variations in lncRNA regions associated with male infertility. Then, we performed an in silico analysis investigating the lncRNAs that are reported in many subtypes, such as azoospermia, teratozoospermia, etc., exploring the affected pathways and molecular mechanisms that contribute to male infertility. With this approach, we aim to provide a comprehensive summary of the lncRNAs involved in male infertility, which will be of particular interest to the life science community and will help biologists unravel the role of lncRNA in the mystery of male infertility.

## 2. Methods

We conducted this systematic review on lncRNAs associated with male infertility following the best-practice Preferred Reporting Items for Systematic Reviews and Meta-Analyses (PRISMA) guidelines [17], using the protocol registered in the International Prospective Register of Systematic Reviews (PROSPERO) (ID: CRD42022356032).

The PICO (Population, Intervention, Comparison, Outcomes) strategy was used to develop the research question and define our inclusion and exclusion criteria as follows: P—adult men; I—study of long noncoding RNAs; C—men with and without male infertility; O—association with sperm parameters (sperm quality, quantity, etc.).

### 2.1. Search Strategy

An extensive search for peer-reviewed articles on male infertility and long noncoding RNAs (lncRNAs) was performed on 1 June 2022, using Pubmed and Web of Science databases.

We operationalized our search for the term male infertility by integrating the methodology of two systematic reviews on topics associated with male infertility [18,19] as follows: “semen” OR “sperm” OR “spermatozoa” OR “oligozoospermia” OR “asthenozoospermia” OR “teratozoospermia” OR “male subfertility” OR “male infertility” OR “spermatogenesis” OR “male infertil*” OR “infertile male*” OR “infertile men” OR “male subfertil*” OR “male steril*” OR “sperm*” OR “seminal.” For the search on Pubmed, we also used the MeSH (medical subject headings) term “Male Infertility” with the Boolean “OR” to join the searches. Similarly, for the term long noncoding RNAs, following Quan et al. (2018) [20] and Tian et al. (2018) [21] and based on their previously validated searches, we also operationalized our search to identify all the keyword variants as: “long noncoding RNA” OR “long noncoding RNAs” OR “long non coding RNA” OR “long non coding RNAs” OR “long non-coding RNA” OR “long non-coding RNAs” OR “long ncRNA” OR “long ncRNAs” OR “lncRNA” OR “lncRNAs” OR “lincRNA” OR “lincRNAs” OR “long intergenic non-coding RNA” OR “long intergenic non-coding RNAs” OR “long intergenic non coding RNA” OR “long intergenic non coding RNAs” OR “long untranslated RNA” OR “long untranslated RNAs” OR “long non-protein-coding RNA” OR “long non-protein-coding RNAs” OR “long non protein coding RNA” OR “long non protein coding RNAS” OR “long intergenic non-protein coding RNA” OR “long intergenic non-protein coding RNAs” OR “long intergenic non protein coding RNA” OR “long intergenic non protein coding RNAs.” The two terms (male infertility and long noncoding RNAs) were combined with the “AND” connector. In addition, for the Pubmed search, we applied the title/abstract fields in our search, whereas for the Web of Science search, we used the topic field.

After both searches, the articles were collected and duplicates were removed using automation tools (Zotero Citation Manager software, https://www.zotero.org/, accessed on 17 September 2022).

### 2.2. Inclusion/Exclusion Criteria

Next, we applied a series of inclusion and exclusion criteria for the articles identified. More specifically, articles were included if they: (i) were written in English, (ii) included human subjects (adults), and (iii) included participants diagnosed with male infertility (based on the results of the seminogram). Studies were excluded if they were conducted on animals or plants, as well as reviews, meta-analyses, and other types of publications that did not report original data. In addition, articles that did not provide accurate and sufficient data and studies that did not have an appropriate control group or that used mixed samples of fertile and infertile males, thus, not allowing for comparison between fertile and infertile males as defined by the PICO strategy, were also excluded. Studies on other types of ncRNAs, such as piRNAs or circRNAs, were also excluded because the present systematic review focuses on lncRNAs and their association with male infertility. Finally, studies on Klinefelter syndrome were excluded. Although it is associated with male infertility, the mechanism that leads to it is completely different, as it is caused by a chromosomal abnormality (extra X chromosome). The full list of inclusion/exclusion criteria is provided in Table 1.

Based on the above criteria, the articles were subsequently screened manually by title, keywords, and abstract for eligibility. At the eligibility stage, the full article was used to select the final sample of articles included in the present systematic review according to our inclusion and exclusion criteria. One investigator (M.K.) reviewed the articles during the screening and eligibility stages, and in case of uncertainty, a second investigator (Z.M.) decided after discussion on the final inclusion or exclusion of studies in the present systematic review.

### 2.3. Extraction and Analysis

For the studies included in this systematic review, we collected the following information from the full text: (i) characteristics of studies and samples (surname of the author, year of publication, country of origin, biological material, participants), (ii) lncRNAs associated with male infertility and particular subtypes (differentially expressed lncRNAs and variants found on lncRNAs regions), (iii) interactions between lncRNAs-miRNAs, (iv) genes and pathways affected or mechanism of action of lncRNAs, (v) impact–association with sperm parameters (sperm quality and quantity according to the seminogram results), and (vi) main methodology used. All data were extracted manually from the full text (investigator: M.K.) and any discrepancy was resolved by discussion with the other two investigators (T.S., Z.M.).

In case of data presented only in graphs or diagrams but not in text, these data were extracted from the published graphs. In case of missing data, these studies were included in the systematic review including only the data provided by the authors. This is a limitation of the present systematic review discussed in the Discussion section.

### 2.4. In Silico Analyses: Identification of Dysregulated lncRNAs, Target Genes and Functional Enrichment Analysis

The results of the analysis were categorized according to different subtypes of male infertility. Dysregulated lncRNAs on at least three subtypes of male infertility which had been reported in multiple studies were selected for further evaluation and in silico analysis. Dysregulated lncRNAs are considered lncRNAs with a differential expression between male infertility patients and normozoospermic individuals. The potential target genes of these lncRNAs were searched using ENCORI (The Encyclopedia of RNA Interactomes) [22]. In order to better understand the biological relevance of lncRNA target genes and to investigate their role in male infertility, functional enrichment analysis was performed using Gene Ontology (GO) [23,24] and KEGG pathways [25]. Statistical significances were reported after false discovery rate (FDR) corrections for multiple comparisons.

## 3. Results

### 3.1. Study Selection

**Figure 1:**
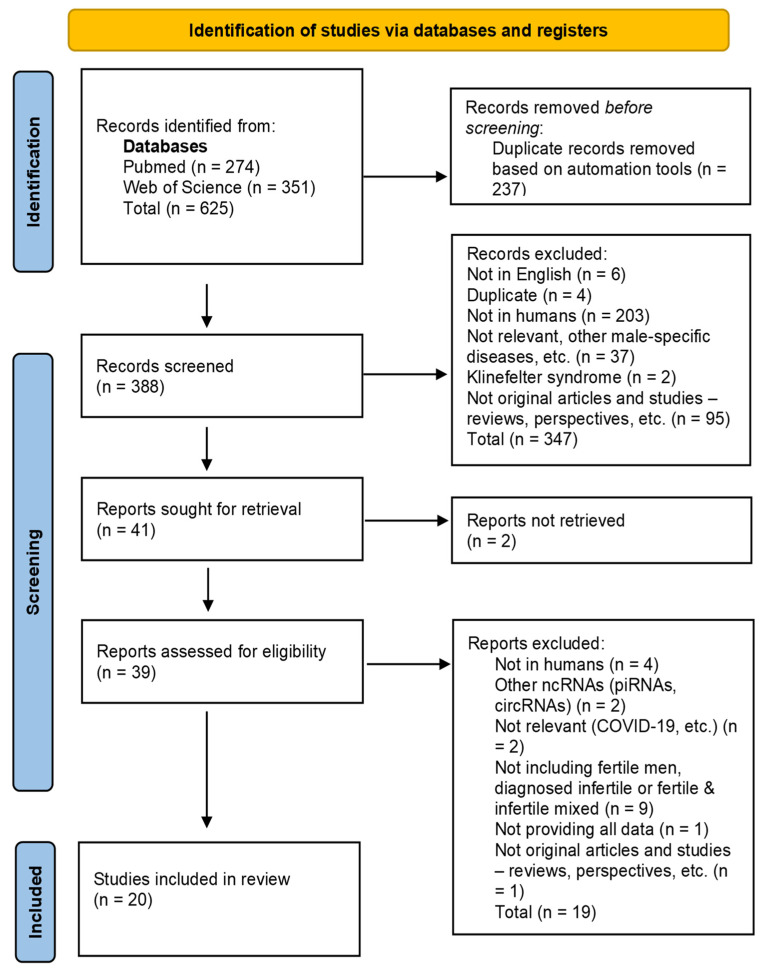
Preferred Reporting Items for Systematic Reviews and Meta-Analyses (PRISMA) diagram of the article selection process.

As observed in the PRISMA flow diagram (Figure 1), our initial search identified a total of 274 articles in PubMed and 351 articles in Web of Science, which were imported into the Zotero reference manager. Of these 625 articles, 237 were identified as duplicates, leaving a total of 388 articles for the screening and eligibility stages.

Based on the inclusion and exclusion criteria described above, the articles were subsequently screened manually by title, keywords, and abstract for eligibility. Of the 388 records screened, 6 were not in English, 203 studied animals or plants, 37 were not relevant to male infertility, 2 studied Klinefelter syndrome, and 95 were articles with non-original data (reviews, perspectives, etc.). Additionally, 4 duplicate articles not previously identified by automated tools were also removed, leaving a total of 41 articles for retrieval. We were unable to find the full text for 2 articles, resulting in 39 articles for eligibility. At the eligibility stage, we excluded 4 studies which did not include human subjects and 2 articles studying specific types of ncRNAs which are not in the scope of this review (circRNAs, piRNAs, etc.). We also further excluded 2 non-relevant studies, 1 study because it provided insufficient data, and 9 studies which used mixed samples of fertile and infertile males, did not include fertile males as controls, or did not include diagnosed infertile males and used other criteria for evaluation of fertility status. One more article was also excluded, as it was a perspective and did not present original data. Finally, a total of 20 articles remained to be included in the present systematic review [26,27,28,29,30,31,32,33,34,35,36,37,38,39,40,41,42,43,44,45].

### 3.2. Study Characteristics (Included Studies)

**Figure 2:**
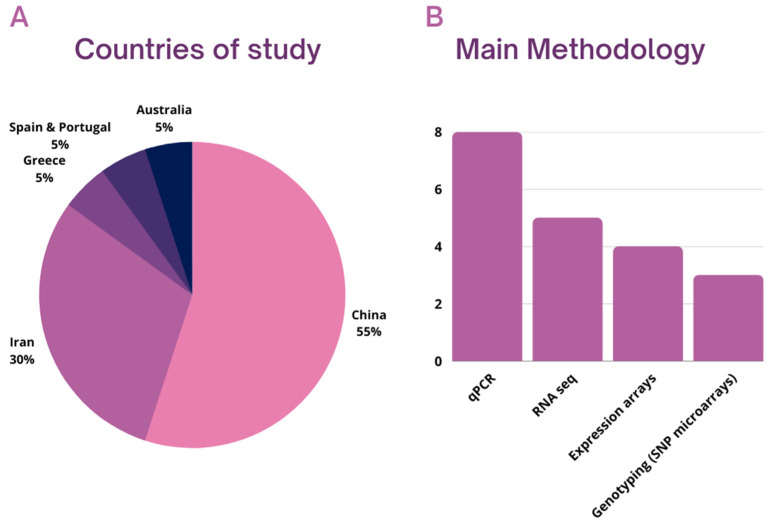
Countries from which the studies originated (**A**), and main methodology used for the investigation of lncRNAs on male infertility (**Β**).

**Figure 3:**
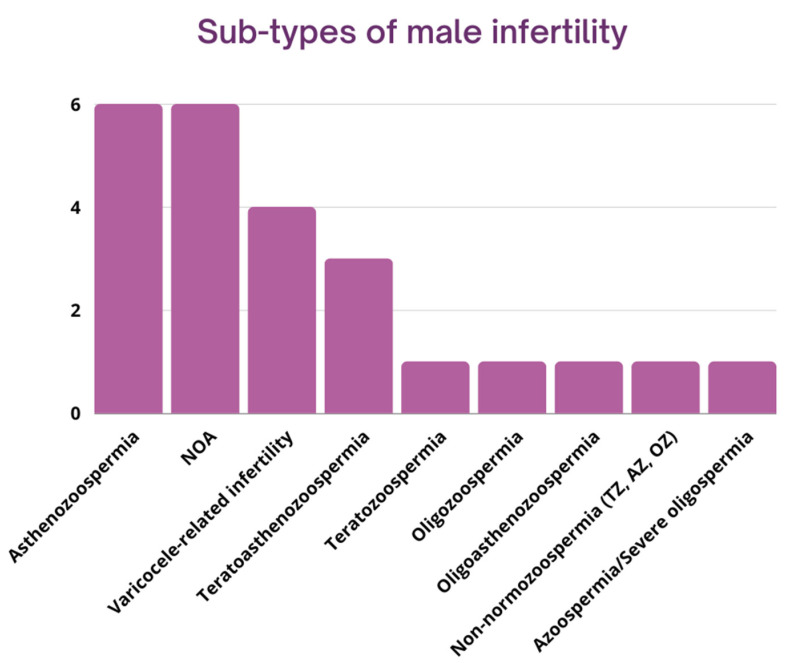
Studies included in the systematic review categorized by sub-type of male infertility; NOA: non-obstructive azoospermia; TZ: teratozoospermia; AZ: asthenozoospermia; OZ: oligozoospermia.

Twenty studies were included in the present systematic review. All studies included originated from 2015 or later, and their number increased after 2020. Of the 20 papers in the analytical sample, 11 articles originated from China, 6 from Iran, and 1 from each of the following countries: Australia, Greece, Spain, and Portugal (Figure 2A). The most widely used methodology was quantitative PCR (*n* = 8), followed by RNA sequencing (*n* = 5) and microarrays/expression arrays (*n* = 4). Some studies also used genotyping and SNP arrays to investigate the role of DNA variants and lncRNAs on male infertility (*n* = 3) (Figure 2B). The tissues analyzed were semen (*n* = 14), testes (*n* = 4), and blood (*n* = 2). Regarding the semen samples, some of the studies investigated only the seminal plasma and/or the extracellular vesicles, such as exosomes, found on them (*n* = 3). Regarding information about the human subjects included in the studies, approximately half of them (*n* = 9) included men aged between 18–42 years old. A few also included older men (<60 years old) (*n* = 3). A significant number of studies, however (*n* = 8), reported no information about the age of the individuals studied. It should also be noted that only a few studies provided detailed information about the general health status of the individuals (body mass index, hormonal status, infections, other diseases, previous cancer treatment, etc.), but at least some of them (*n* = 12) stated clearly that individuals with underlying diseases, infections of the urogenital tract, injuries, heavy smokers, or men reporting excessive alcohol or drug consumption were excluded. Next, we disaggregate the studies according to the different subtypes of male infertility, as presented in Figure 3. We briefly describe each of these in turn in the next section, starting with azoospermia. It should be noted that some studies appear in many subsections, as they studied the role of lncRNAs on many subtypes of male infertility simultaneously.

The summary characteristics of the included studies are presented in Table S1.

### 3.3. Subtypes of Male Infertility

#### 3.3.1. Azoospermia (*n* = 6)

Azoospermia is described as the complete absence of sperm in the ejaculate [46]. Azoospermia can be also distinguished into two broad categories: (a) obstructive azoospermia, when the spermatogenesis process is unaffected, but a blockage exists leading to infertility, and (b) non-obstructive azoospermia (NOA) that is due to impaired or absent spermatogenesis [46,47]. It can be classified histologically into three subtypes (hypospermatogenesis, maturation arrest, and Sertoli cell-only syndrome) [47]. Sertoli cell-only syndrome, also known as Del Castillo syndrome or germ cell aplasia, is defined by the complete absence of germ cells and the presence of only Sertoli cells, which normally support and nurture the immature sperm and line the seminiferous tubules [48]. It is considered the most common subtype of non-obstructive azoospermia [48,49]. In contrast, in hypospermatogenesis (Hypo), all stages of germ cells observed during spermatogenesis are present (spermatogonia, spermatocytes, spermatids) but their number is decreased [50]. A testicular biopsy is usually required to evaluate the samples and diagnose the above conditions [51].

Studies regarding azoospermia usually investigate the differential expression of lncRNAs among patients and healthy males (Table S2). Lü et al. (2015) [26], using microarray technology, identified 757 and 2370 differentially downregulated and 475 and 163 upregulated lncRNAs in NOA patients with meiotic arrest (MA) and hypospermatogenesis (Hypo), respectively. Among them, the lncRNA *NLC1-C* was downregulated in the cytoplasm and accumulated in the nucleus of spermatogonia and primary spermatocytes of patients, suggesting a role in the early stages of spermatogenesis, regulating germ cell proliferation and death. The *HOTTIP* lncRNA was also found to be downregulated in patients with NOA in the present study. Su et al. (2019) [39] confirmed also that *HOTTIP* is downregulated in testicular tissues of Hypo patients and plays a role in cell proliferation, potentially affecting spermatogenesis with this mechanism. It is highly expressed in the testicular embryonal carcinoma cell line, according to the experiments of the researchers. Its localization in both the cytoplasm and the nucleus also indicates that it is associated with male infertility by acting on transcriptional and post-transcriptional levels.

Some studies (*n* = 2) have also studied the expression profiles of lncRNAs, but in addition, they investigate the mRNA-miRNA-lncRNA interaction networks with a potential role in azoospermia. Bo et al. (2020) [29] detected the competing endogenous RNA (ceRNA) networks of lncRNAs. They found 187 differentially expressed lncRNAs (13 upregulated and 174 downregulated) between NOA patients and controls (Table S2), as well as a few differentially expressed lncRNAs, which were only found between controls and a subgroup of NOA patients with Sertoli cell-only syndrome (Table S2). Investigation of target genes involved in ceRNA networks led researchers to the conclusion that testicular lncRNAs regulate spermatogonial cell proliferation or spermatocyte meiosis. Similarly, Sabetian et al. (2022) [33] found that differentially expressed lncRNAs, miRNAs, and mRNAs in patients with idiopathic NOA constructed mRNA-miRNA-lncRNA interaction networks. A total of 10 lncRNAs were identified to be downregulated in NOA patients in comparison with healthy individuals (Table S2). Their study suggests that disrupted interactions of lncRNAs–miRNAs–mRNAs can lead to spermatogenesis arrest and male infertility.

Studies also propose that lncRNAs have the potential to be used for diagnostic purposes (*n* = 1). Xie et al. (2020) [27] examined the expression profiles of lncRNAs found on extracellular vesicles in the seminal plasma between healthy males and NOA patients that had been diagnosed with Sertoli cell-only syndrome, and in all of them, no testicular spermatozoa were obtained after microdissection testicular sperm extraction (mTESE). Finally, they report a panel of nine differentially expressed lncRNAs (Table S1) that can be used to predict the presence of testicular spermatozoa in patients with NOA. All of the lncRNAs exert testis-specific expression and, in addition, the authors highlight that this panel has an advantage even in comparison with other models of diagnosis based on serum hormone levels or seminal plasma exosomal miRNAs. All of the differentially expressed lncRNAs found in this study are presented in Table S3.

**Figure 4:**
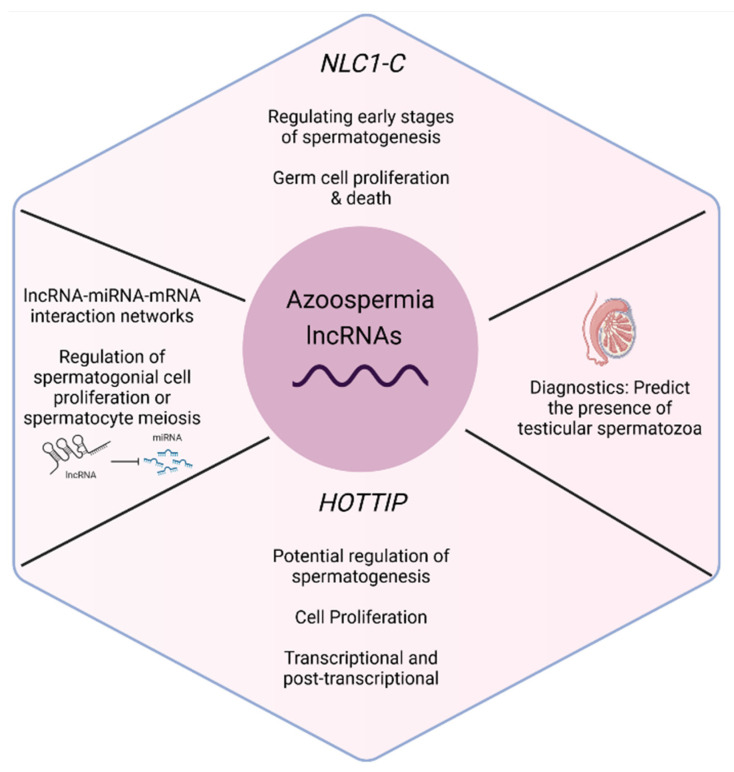
Individual lncRNAs with a role in spermatogenesis and azoospermia according to the studies included in the present systematic review. Figure made in Biorender.com (accessed on 17 September 2022).

A summary of the lncRNAs with a potential role in azoospermia, according to publications studying the expression profiles of lncRNAs and study characteristics, is presented in Table S2. Individual lncRNAs with a role in spermatogenesis, and especially in azoospermia, according to the above studies, are also summarized in Figure 4.

#### 3.3.2. Asthenozoospermia (*n* = 6)

Asthenozoospermia is characterized by reduced sperm motility, and it is arguably the main cause of male infertility [52].

As in the studies on azoospermia, studies that investigated the role of lncRNAs on asthenozoospermia analyzed their expression profiles (Table S4). Zhang et al. (2019) [31] identified 6393 upregulated and 3486 downregulated lncRNAs in asthenozoospermic men compared with males with normal semen parameters. They especially focused on three sperm/testis-specific/enriched lncRNAs which were upregulated in asthenozoospermia patients (Table S4). The expression levels of all three lncRNAs were negatively associated with sperm progressive motility, indicating a potential regulatory role. In addition, bioinformatics tools were used to predict the genes targeted by differentially expressed (DE) lncRNAs, and these were involved, among others, in cellular macromolecule biosynthetic processes, spermatogenesis, male gamete generation, cytoplasmic transport, protein processing in the endoplasmic reticulum, hippo signaling pathways, fatty acid metabolism, and the RNA metabolic process.

Some studies on asthenozoospermia also focus from the beginning of their investigation on specific lncRNAs, based on previous evidence (*n* = 4). Zhang et al. (2015) [35] identified that *HOTAIR* was downregulated in patients with asthenozoospermia in comparison with healthy individuals. *HOTAIR* is a well-studied lncRNA which, according to several studies, promotes the development of tumors. The *HOTAIR* expression level was also found to be positively correlated with sperm motility. With functional experiments in cell lines, Zhang et al. (2015) [35] proved that *HOTAIR* can regulate Nrf2 expression by mediating histone H4 acetylation in the Nrf2 gene promoter, a transcription factor that protects spermatozoa from oxidative stress. Therefore, the reduction of *HOTAIR* and the subsequent reduction of Nrf2, which has a protective role in normal cells, enhances oxidative stress, resulting in a negative impact on sperm motility and the presence of asthenozoospermia. Similarly, Saberiyan et al. (2020) [43] explored the association between *ANO1-AS2* lncRNA, a gene that is located near it (*ANO1*, anoctamin), and asthenozoospermia. The expression levels of the lncRNA were significantly upregulated in patients with asthenozoospermia in comparison with healthy males, and were negatively associated with sperm motility. Furthermore, *ANO1* gene expression was downregulated, and its expression was positively correlated with sperm motility. Finally, the authors suggest that *ANO1-AS2* can affect sperm motility by downregulating *ANO1* expression via interaction with its promoter. These findings shed light on the molecular mechanism of asthenozoospermia, as *ANO1* (also known as *TMEM16A*) is an ion channel with an important function as a member of the transmembrane system [53]. In addition, it is required for the function and membrane expression of *CFTR* [54]. Deregulation of these two can alter the levels of bicarbonate (HCO_3_) that are required for sperm motility [55]. Kamel et al. (2022) [36] chose to investigate the role of another lncRNA in asthenozoospermia. More specifically, *CFAP44-AS1*, an antisense lncRNA of the *CFAP44* gene that exerts testis-specific expression, was considered a good candidate, as *CFAP44* encodes a protein located in the flagellar axoneme with an important role in the formation and function of the flagella and cilia [56]. It was found that both *CFAP44* and *CFAP44-AS1* were downregulated in patients with asthenozoospermia, and a positive correlation was also observed between *CFAP44* and *CFAP44-AS1* expression, as well as between both genes’ expression and sperm motility [36]. Similarly, Saberiyan et al. (2021) [30] studied the expression of *LINC00574* and *TCTE3,* a gene with an important role in flagellum formation. *TCTE3* was downregulated in asthenozoospermic men, while *LINC00574* levels were increased in asthenozoospermic patients in comparison with healthy males. A positive correlation was observed between *TCTE3* and *LINC00574* expression, and between *TCTE3* expression levels and sperm motility. Furthermore, a negative self-regulating mechanism occurs between these two, affecting sperm motility.

Seminal plasma exosomes can also provide valuable information about the role of lncRNAs on asthenozoospermia. Lu et al. (2020) [41] found 4228 differentially expressed genes: 995 known differentially expressed lncRNAs, 2338 differentially expressed mRNAs, and 11,706 novel differentially expressed lncRNAs between asthenozoospermia and healthy men. Regarding lncRNAs, 656 were found to be upregulated and 339 downregulated in asthenozoospermia, compared to the normal control group. The full list of DE lncRNAs found in this study is presented in Table S3.

**Figure 5:**
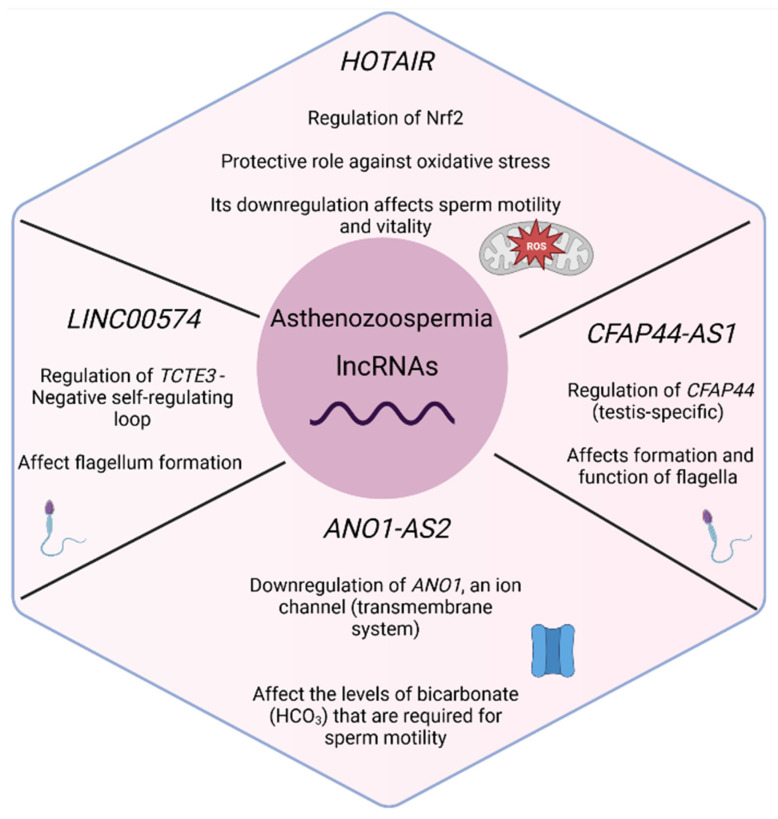
Individual lncRNAs with a role in spermatogenesis and asthenozoospermia according to the studies included in the present systematic review. Figure made in Biorender.com.

A summary of the lncRNAs with a potential role in asthenozoospermia, according to publications studying the expression profiles of lncRNAs, as well as study characteristics is presented in Table S4. Individual lncRNAs with a role in spermatogenesis and, more specifically, in asthenozoospermia, as well as their mechanism of action according to the above studies, are also presented in Figure 5.

#### 3.3.3. Teratozoospermia (*n* = 1)

Teratozoospermia is another sub-type of male infertility characterized by spermatozoa with abnormal morphology. It is considered a heterogenous disorder, since it includes many abnormal sperm phenotypes which are characterized by, defects on the head, neck, midpiece, or tail of sperm, among others [57].

The only study investigating the role of lncRNAs in teratozoospermia aimed to identify long noncoding RNAs acting as competing endogenous RNAs (ceRNAs). They found 101 differentially expressed lncRNAs (68 upregulated and 33 downregulated in the teratozoospermic group) and 1722 differentially expressed mRNAs between teratozoospermic and normozoospermic men. The full list of differentially expressed lncRNAs on teratozoospermia reported in this study is presented in Table S3. Then, the researchers constructed ceRNA networks with 26 key lncRNAs, 33 microRNAs, and 133 mRNAs. After GO and KEGG analysis, it was also found that differentially expressed lncRNAs were associated with transferring phosphorus-containing groups and complexes of histone methyltransferases, methyltransferases, PcG proteins, and serine/threonine protein kinases highlighting important processes for teratozoospermia [45].

Study characteristics, as well as the most important lncRNAs reported to have a potential role in teratozoospermia, are presented in Table S5.

#### 3.3.4. Oligozoospermia (*n* = 1)

Oligozoospermia is another subtype of male infertility associated with low sperm count. Only one study was identified that explored the specific lncRNAs associated with oligozoospermia.

More specifically, Sun et al. (2021) [37] examined the expression profile of lncRNAs in oligozoospermia, and found 2364 differentially expressed lncRNAs between oligozoospermic and healthy males. A total of 464 lncRNAs were downregulated, while 1900 lncRNAs were upregulated (Table S3). GO and KEGG analysis for the differentially expressed mRNAs and the target genes of differentially expressed lncRNAs revealed some key pathways for oligozoospermia, such as PERK-EIF2 pathway, induced ER stress, accumulation of unfolded proteins in sperm ER, oxidative stress, and sperm cell apoptosis. Thus, as a possible mechanism of oligozoospermia, it is suggested that the accumulation of unfolded protein due to mutations or impaired folding can lead to endoplasmic reticulum stress, as well as ER stress-triggered oxidative stress that results in the apoptosis of sperm cells.

The top differentially expressed lncRNAs on oligozoospermia and study characteristics are presented in Table S6.

#### 3.3.5. Oligoasthenozoospermia (*n* = 1)

Oligoasthenozoospermia combines two sperm defects, and is characterized by a decreased number of motile spermatozoa and a reduced total number of spermatozoa.

Only one study (Table 2) was identified that investigated the relationship between lncRNAs and oligoasthenozoospermia. As in the case of asthenozoospermia, Zhang et al. (2015) [35] studied the role of *HOTAIR* in oligoasthenozoospermia, with the same results. The expression of *HOTAIR* was decreased in patients, as was the expression of Nrf2, and oxidative stress was also found to be the main cause of oligoasthenozoospermia. However, it should be noted that *HOTAIR* was positively correlated with sperm motility and vitality, but not with sperm concentration. That means that the downregulation of *HOTAIR* leads to the downregulation of Nrf2, as well as the enhancement of oxidative stress that results in apoptosis and affects sperm motility and vitality. Thus, *HOTAIR* can be considered a protective lncRNA for spermatozoa, preventing them from oxidative damage.

#### 3.3.6. Teratoasthenozoospermia (*n* = 3)

Teratoasthenozoospermia (TAZ) is also a combination of sperm defects, as in this case, a low percentage of spermatozoa with normal morphology as well as a reduced number of spermatozoa was observed in semen samples.

As in the studies on asthenozoospermia, studies on teratoasthenozoospermia focus on specific lncRNAs (Table 3). Saberiyan et al. (2020) [43] studied *ANO1-AS2* and *ANO1,* and found that *ANO1-AS2* was upregulated in TAZ patients while *ANO1* was downregulated. The expression of *ANO1-AS2* was also found to be inversely correlated with sperm morphology, while *ANO1* was positively correlated with it. Therefore, it is suggested that the interaction between ANO1-AS2 and *ANO1* plays an important role in TAZ. This is not surprising, as *ANO1* also interacts with other genes that play a role in male infertility, and especially sperm motility and morphology [58]. Furthermore, since mutations in the *CFAP44* gene are associated with morphological defects in the sperm tail [56], Kamel et al. (2022) [36] also studied the expression of *CFAP44* and *CFAP44-AS1* in patients with asthenoteratozoospermia. As in asthenozoospermia, *CFAP44* and *CFAP44-AS1* were both downregulated in patients with asthenoteratozoospermia and positively correlated with sperm motility and normal sperm morphology.

It should also be noted that Saberiyan et al. (2020) [43] also investigated the role of *LINC00574* in TAZ, but they did not identify any difference in its expression between patients with TAZ and controls.

#### 3.3.7. Varicocele-Related Male Infertility (*n* = 4)

Varicocele (VC) is a vascular disease characterized by abnormal enlargement of the pampiniform plexus veins, which are found within the scrotum. The varicocele is associated with reduced sperm quality and can result in male infertility [59]. It is estimated that 40% of men with male infertility are finally diagnosed with varicocele [60]. Though it is considered a highly treatable cause of male infertility, the molecular mechanisms involved have not been fully revealed [61].

As in other sub-types of male infertility, studies regarding varicocele-related infertility focus on particular lncRNAs (Table 4). LncRNA growth arrested DNA damage-inducible gene 7 (*GADD7*) is considered a well-studied lncRNA, as experiments prove that its complex interactions with Cdk6, among others, regulate oxidative stress-mediated cell death [62]. Oxidative stress has been associated with varicocele; thus, Zhao et al. (2017) [40] explored the role of *GADD7* on varicocele-related infertility. *GADD7* was found to be upregulated in patients with varicocele, and its expression was negatively correlated with sperm count, an important semen parameter. Further experiments in cell lines also confirmed its contribution to male infertility by inhibiting cell proliferation and promoting cell apoptosis. The authors suggest that *GADD7* may exert its effect by upregulating the pro-apoptotic regulator Bax and downregulating the anti-apoptotic regulator Bcl2.

Similarly, Ataabadi et al. (2020) [38] investigated the role of two hypoxia-related lncRNAs in infertile patients with varicocele. Expression levels of *MIR210HG* and *MLLT4-AS1* increased in patients, and a positive correlation with ROS levels as well as a negative correlation with crucial sperm parameters, such as sperm motility and count, were observed. The presence of hypoxia-related elements (HREs) in all of them again highlights the involvement of oxidative stress in the pathogenesis of male infertility, and especially in varicocele-related male infertility. Li et al. (2022) [28] also studied the expression of along noncoding RNA, microRNA210 host gene (*MIR210HG*), in the seminal plasma of varicocele patients (VC), and found even more interesting results. Again, the levels of this lncRNA were also found to be upregulated in patients, distinguishing them from healthy men, but its expression was positively associated with varicocele severity, and decreased in varicocele patients after surgery. The ability of *MIR210HG* to predict a reduction in sperm quality, including sperm parameters such as sperm motility, number, and morphology, which is called dyszoospermia and is observed in varicocele-related male infertility, was also investigated. When comparing varicocele patients with normal semen parameters and varicocele patients with dyszoospermia, researchers observed that *MIR210HG* levels were significantly upregulated in dyszoospermia. Thus, this lncRNA, in addition to contributing to our understanding of the mechanisms involved in male infertility, can also be used to screen patients with varicocele-related male infertility from healthy males, and can act as a biomarker for predicting the severity of varicocele and the presence of varicocele-associated dyszoospermia.

Similarly to the above studies, Sanei-Ataabadi, Mowla, and Nasr-Esfahani, (2020) [34] decided to focus on *SLC7A11-AS1,* which was upregulated in patients with varicocele-related infertility. A positive correlation was also observed between its expression and ROS levels, while a negative correlation was found with sperm parameters such as sperm count and motility. It appears that *SLC7A11-AS1* can lead to male infertility through the downregulation of *SLC7A11*, a gene that plays a role in maintaining redox homeostasis [63]. With this downregulation, ROS levels increase, leading to oxidative stress and the promotion of cell death.

**Figure 6:**
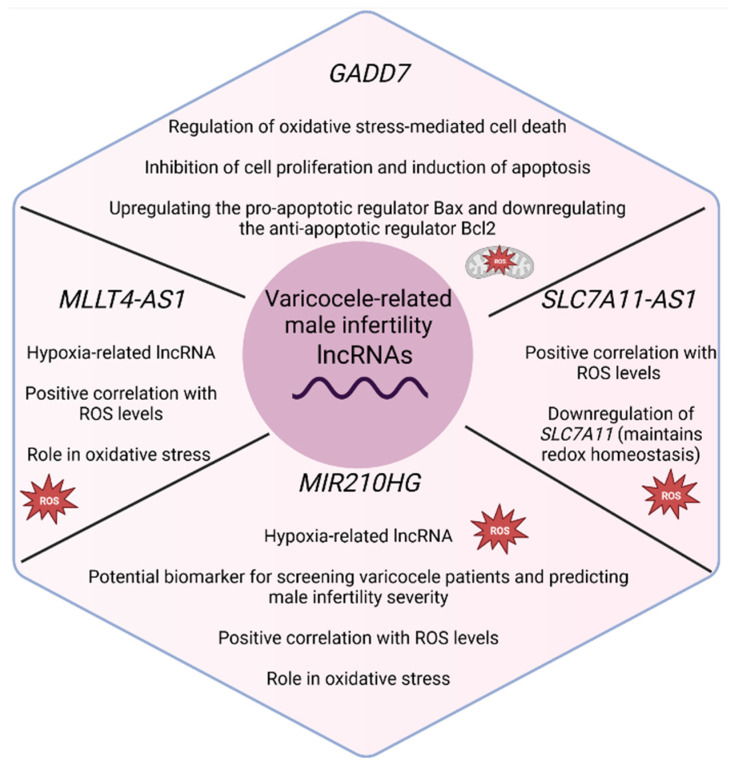
Individual lncRNAs with a role in spermatogenesis and varicocele-related male infertility, according to the studies included in the present systematic review. Figure made in Biorender.com.

Individual lncRNAs with a role in spermatogenesis, and, more specifically, in varicocele-related male infertility, as well as their mechanism of action according to the above studies, are presented in Figure 6.

### 3.4. Interactions between lncRNAs-miRNAs and Putative Target Genes

miRNAs can be considered the most well-studied class of non-coding RNAs [64,65]. They have an average length of 22 nucleotides, and they are important players in the regulation of gene expression. They usually function post-transcriptionally, as they interact with the 3′ untranslated (UTR) region of target mRNAs, affecting their stability and leading to translational repression [65]. Studies show that they can affect the expression of hundreds of target genes through their interactions [65,66].

miRNAs regulate many important processes, including spermatogenesis, and thus have an impact on male infertility [67,68]. They are found in spermatozoa and many other cell types in the testes, such as Leydig cells, Sertoli cells, and spermatogonia [69]. More interestingly, specific miRNAs are expressed in every step of spermatogenesis to coordinate this complex process [64]. Different expression patterns of miRNAs are also observed between normozoospermic and non-normozoospermic men [69]. The important role of miRNAs in spermatogenesis has been proven by many experiments in mouse models, as it was observed that cell-specific deletion of Dicer, which is essential for miRNAs biosynthesis, disrupts the differentiation of male germline [70] and leads to spermatogenic failure [71,72]. Individual miRNAs also regulate meiosis and lead to infertility, according to findings in knockout mouse models [67,73,74]. Furthermore, their role in other biological processes, such as regulation of the cell cycle, cell differentiation, and apoptosis, is associated with an impact on male infertility as well [67]. Characteristic examples are hsa-miR-34b-3p, which regulates male meiosis via the E2 factor-retinoblastoma protein (E2F-pRB) pathway, and hsa-miR-132-3p, which is associated with cell cycle progression through *MYC* activation [66]. Therefore, the impaired biosynthesis of miRNAs, or mutations that affect their interactions with their targets, can lead to the deregulation of all of the above processes, which has an impact on male fertility.

Many studies also suggest that these can be used as biomarkers in male infertility, because they are found in seminal fluid and have specific expression patterns [75]. There are several review articles summarizing their important role in male infertility or spermatogenesis and reporting miRNAs that are deregulated in specific subtypes of male infertility, or miRNAs that could serve as prognostic and diagnostic biomarkers [64,67,69,74,76].

It has been reported that lncRNAs and miRNAs interact with one another, affecting gene expression and exerting a regulatory role [77,78]. Many types of interactions have been reported. More specifically, some lncRNAs are degraded after interactions with miRNAs, serve as sponges or decoys for microRNAs, or compete with miRNAs for binding to mRNAs [77]. Deregulation of these interactions can affect many processes and even lead to diseases [13,79,80]. Among the identified papers which investigate the role of lncRNAs in male infertility, a few of them also reported lncRNA–miRNA interactions. These interactions were confirmed experimentally or predicted based on bioinformatics tools.

The three studies reporting experimentally validated lncRNA–miRNA interaction networks are those performed by Lü et al. (2015) [26], Su et al. (2019) [39], and Bo et al. (2020) [29] (Table 5). Lü et al. (2015) [26] used cell lines and found that *NLC1-C* has a regulatory mechanism that is dysregulated in patients with NOA. In healthy males, in the nucleus, *NLC1-C* inhibits miR-320a and miR-383 transcripts by binding to an RNA-binding protein, the nucleolin. When *NLC1-C* and precursor miR-320a/383 are exported to the cytoplasm, it is observed that there, precursor miR-320a/383 is processed into its mature form and targets *NLC1-C,* resulting in the regulation of spermatogenesis. In contrast, researchers found that in patients with NOA, *NLC1-C* is downregulated in the cytoplasm, and accumulates in the nucleus of spermatogonia and primary spermatocytes. This accumulation is associated with male infertility, as, by binding to nucleolin, it represses miR-320a and miR-383, which regulate cell proliferation and apoptosis, leading to the hyperactive proliferation of spermatogonia and primary spermatocytes. Similarly, Su et al. (2019) [39] showed that *HOTTIP* is involved in the pathogenesis of azoospermia as a ceRNA which acts as a sponge for miR-128-3p. In Hypo patients, downregulation of *HOTTIP* led to inhibition of cell proliferation. This was not a surprise, as miR-128-3p has been indicated in previous studies to have a role in the inhibition of cell proliferation, but further investigation suggested that its target gene may be *HOXA13,* which contributes to the proliferation of tumor cells. Therefore, this competitive binding between lncRNA-miRNA can also lead to positive regulation of *HOXA13* expression, resulting in male infertility.

Bo et al. (2020) [29] used a different approach. At first, they constructed competing endogenous networks of lncRNAs by using microarray datasets of NOA males and healthy males. After detecting differentially expressed lncRNAs and mRNAs, they predicted the miRNA interactions which would construct the lncRNA–miRNA–mRNA networks. They also used only miRNAs found to be differentially expressed in patients with NOA, according to previous studies. Therefore, they found 1269 interaction pairs of miRNAs, lncRNAs, and mRNAs in a complex regulatory network with 572 nodes and 1296 edges. From all these interactions, they further explored the interactions of a testis-specific lncRNA, *LINC00467*, by performing experiments in cell cultures. Thus, they found that this lncRNA positively regulates the expression of *TDRD6* and *LRGUK* through miR-500-3p. Both of these genes are associated with the spermatogenesis process.

In the studies included in the present systematic review, many more lncRNAs–miRNAs interactions that were detected through prediction models have been reported. Zhou and Wang (2020) [45] studied competing endogenous networks in teratozoospermia and found interactions between 26 key lncRNAs, 33 microRNAs, and 133 mRNAs (Table S7). These interactions were also predicted with bioinformatic tools after the identification of differentially expressed mRNAs and lncRNAs. The differentially expressed mRNAs targeted by lncRNAs–miRNAs pairs were enriched in embryonic skeletal system development and cytokine–cytokine receptor interactions, as well as in pathways involving nicotinamide adenine dinucleotide (NADH), such as oxidoreductase activity, electron transfer activity, and NADH dehydrogenase activity. It should also be noted that Bo et al. (2020) [29] also constructed competing endogenous networks of lncRNAs in NOA patients, as reported earlier. Among them, the following are reported: *TUSC7*–miR-34-5p–*GSG1*, *TMEM239*–miR-34-5p–*PWRN2*.

Sabetian et al. (2022) [33] also conducted an extensive study in order to identify dysregulated interactions between lncRNAs–miRNAs–mRNAs in patients with idiopathic NOA. They found 74 mRNAs, 14 miRNAs, and 10 lncRNAs which were differentially expressed between the testicular tissues of NOA patients and healthy individuals. All lncRNAs were found to be downregulated while all miRNAs were upregulated. They report that nine of these lncRNAs interact with two miRNAs which were both upregulated in NOA patients (miR-27b-3p, miR-509-3-5p). The target genes of these two miRNAs are polo-like kinase 1 (*PLK1*) and Cysteine-rich secretory protein2 (*CRISP2*). *PLK1* has a crucial role in the spermatogenesis process, and *CRISP2* contributes to the regulation of sperm flagellar motility, the acrosome reaction, and sperm–egg fusion [81]. It should be noted that although the lncRNAs–miRNAs interactions were predicted with bioinformatics tools, only reciprocal miRNA–mRNA interactions validated experimentally were used for the construction of the above networks, presented in Table S7.

Finally, Lu et al. (2020) [41] also analyzed the expression profile of lncRNAs found in seminal plasma exosomes of patients with asthenozoospermia. Based on the differential expression of lncRNAs and mRNAs, they predicted lncRNAs–miRNAs–mRNAs networks using bioinformatics. The detailed regulatory lncRNA–miRNA–mRNA network included 11 lncRNAs (*LINC00893, AC005034.3, COX10-AS1, MIR497HG, LINC00894, AC015813.1, AP000424.1*, *MIR17HG, LINC00667, LINC00662,* and *SNHG3*), 35 miRNAs, and 59 mRNAs (Table S7). GO and KEGG analyses regarding the differentially expressed mRNAs revealed that most genes regulated by lncRNAs–miRNAs interactions were associated with metabolism, transcription, ribosome, and channel activity, highlighting potential pathways affected in asthenozoospermia.

### 3.5. Exploring Variants on lncRNAs (n = 3)

Genetic variants have been studied extensively in recent years in an attempt to disentangle the molecular mechanisms of complex disorders [82]. Genome-wide association studies (GWAS) that are performed to identify whether any variant is associated with the phenotype of interest can provide especially valuable information about risk variants, protective variants, etc. [83]. As only 10% of variants are mapped on protein-coding regions, many studies also explore variants on non-coding regions that can be used for diagnostic purposes [84], or to shed light on molecular mechanisms of the disease by deregulating complex interactions between non-coding RNAs and other RNAs or proteins [10]. Thus, among the studies analyzed in the present systematic review, a few did not investigate the expression profiles of lncRNAs, but explored variants in long non-coding regions that are associated with male infertility.

Eggers et al. (2015) [32] searched for copy number variations (CNVs) associated with male infertility, and particularly in patients with meiotic arrest. Using microarray technology (Affymetrix GeneChip Human Mapping 500K EA array), they identified 64 CNVs. However, after comparison with healthy individuals, only two CNVs were identified to be specifically found only in infertile men. In the second CNV region, on chromosome 11, an uncharacterized lncRNA transcribed in the human testis was found (*LOC100507205*). The authors conclude that these CNVs could be indicative of regions that play a role in the pathogenesis of male infertility, and could be used as biomarkers for a successful diagnosis.

Kyrgiafini et al. (2020) [42] performed a GWAS involving 159 individuals: 76 normozoospermic, and a mixed sample of 83 non-normozoospermic that were oligozoospermic, asthenozoospermic, teratozoospermic, or a combination of the previous states. At first, they identified single nucleotide polymorphisms (SNPs) associated with male infertility that were found within or near lncRNAs regions. Then, by performing bioinformatics analysis, they predicted lncRNAs–miRNAs interactions. According to the role of the genes targeted by lncRNAs–miRNAs pairs in the spermatogenesis process or other processes involved in fertilization, they concluded that variants found on six lncRNAs (Table S8) are associated with male infertility through their interaction with miRNAs (miR-410-3p, miR-429, miR-190a-5p, miR-181d-5p, miR-181a-5p, miR-34b-3p, and miR-509-3p), as their presence can lead to a disruption of the above mechanism.

Cerván-Martín et al. (2021) [44] also conducted a GWAS in order to validate the effect of six SNPs, which were associated with NOA in the Han Chinese population, on male infertility. They found an association for three of these SNPs after testing on a sample of 674 infertile Iberian men (480 NOA and 194 patients with severe oligospermia) and 1058 healthy individuals. In silico analysis revealed that *CDC42BPA*-rs3000811 led to a decreased expression of *LINC01641,* while a gene located near this lncRNA, *CDC42BPA,* plays a role in cytoskeletal reorganization and has the potential to affect both mitosis and meiosis. Similarly, the LD block, including the *ABLIM1*-rs7099208, seems to affect the expression of a testis-specific lncRNA, *RP11-38C6.2*, as well as *FAM160B1*.

All of the lncRNAs with a potential role in male infertility, according to papers investigating genetic variants described above, as well as study characteristics, are presented in Table S8.

### 3.6. In Silico Analysis

After finding all lncRNAs which have been reported to be differentially expressed in male infertility patients according to the analyzed papers, it was observed that some of them appear in many studies and many sub-types of male infertility. As presented in Table 6, 28 lncRNAs were reported to be dysregulated in patients with male infertility in two sub-types, while 8 lncRNAs were dysregulated in three sub-types and in multiple studies (*SPATA42, LINC00710, RUSC1-AS1, LINC00905, COX10-AS1, HOTAIR, LINC01091,* and *LINC01359*). These 8 lncRNAs were chosen for further evaluation.

It should be noted, however, that regarding the type of regulation (up/downregulation) of the lncRNAs, many studies included in this systematic review did not provide adequate information; thus, information is presented in the following table (Table 6) only when available.

Next, bioinformatics analysis was performed in order to identify putative target genes of the deregulated lncRNAs and biological pathways in which these lncRNAs are involved. For four of the eight lncRNAs, no information was found in the databases about their target genes. The other four lncRNAs *(HOTAIR, RUSC1-AS1, COX10-AS1,* and *LINC01359*) altogether have interactions with approximately 100 genes. *HOTAIR* has the largest number of target genes (80 genes), followed by *COX10-AS1* (17 genes), and both *LINC01359* and *RUSC1-AS1* have one target gene. Among all target genes, 71 were protein-coding, 23 were miRNAs, 3 were lncRNAs, and 1 was rRNA. No common target genes were observed between the lncRNAs (Table S9).

**Figure 7:**
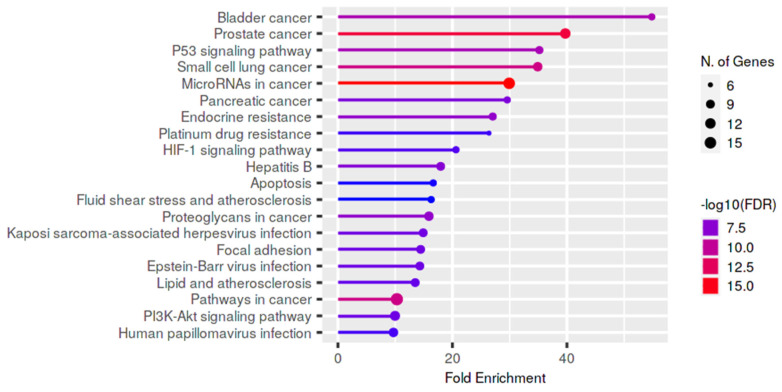
Significant KEGG pathways potentially regulated by the dysregulated lncRNAs in male infertility. The size and color of the dots represent the number of genes and the range of the pathway’s statistical significance, respectively. The *y*-axis represents the KEGG pathways, and the *x*-axis the fold enrichment. The *p*-values were corrected for multiple tests using the false discovery rate (FDR).

In order to explore the functional consequences of the selected deregulated lncRNAs on male infertility, we also performed a functional enrichment analysis of the genes targeted by these lncRNAs using KEGG pathways (Figure 7). Most of the pathways were found to be associated with cancer and the p53 signaling pathway.

**Figure 8:**
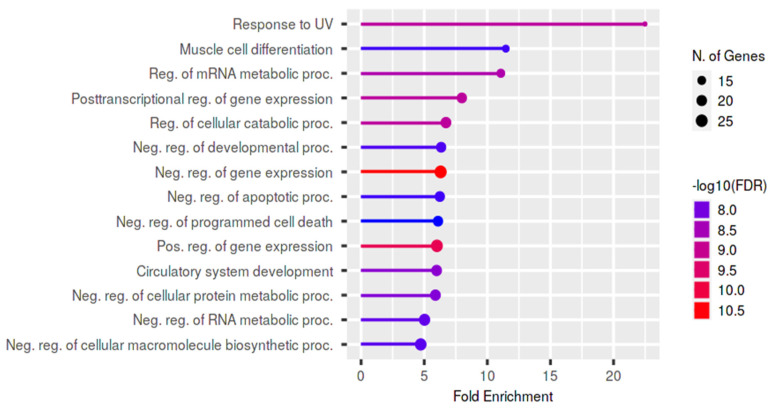
Significant GO Biological Function terms associated with the dysregulated lncRNAs in male infertility. The size and color of the dots represent the number of genes and the range of statistical significance, respectively. The *y*-axis represents the GO terms for biological function, and the *x*-axis the fold enrichment. The p-values were corrected for multiple tests using the false discovery rate (FDR).

Functional enrichment of the target genes was also performed using the GO biological process (Figure 8), in order to obtain more biological information on the potential role of lncRNAs in male infertility.

## 4. Discussion

### 4.1. Main Findings

An expanding body of evidence has indicated the role of lncRNAs in many biological processes [85,86] and diseases [79,87,88]. Our analysis has found evidence that many lncRNAs are differentially expressed between patients with male infertility and normozoospermic men, indicating a regulatory role in fertility. Some of them are deregulated only on specific sub-types of male infertility, suggesting that different mechanisms may be involved in every sub-type. A few of the lncRNAs detected are also deregulated on many sub-types, providing a basis for a common cause of male infertility associated with fundamental cellular processes required for the fertilization process. Furthermore, complex interactions between lncRNAs and miRNAs have an impact on gene expression and can lead to male infertility. However, what may be the most important finding is the fact that these interactions, as well as functional enrichment analysis of the eight lncRNAs that were found to be deregulated on many male infertility sub-types and reported in many studies, show an association with tumor development pathways.

### 4.2. In Silico Analysis: Male Infertility and Cancer

**Figure 9:**
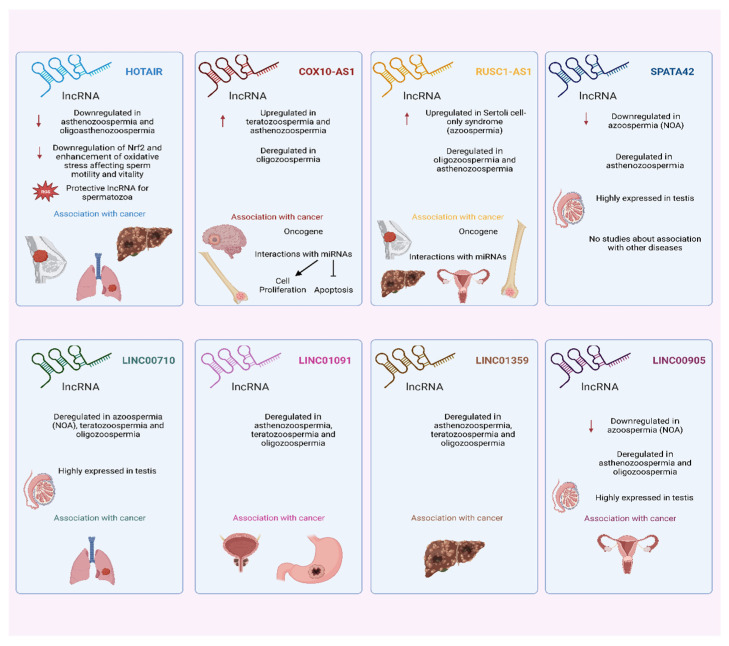
The eight differentially expressed lncRNAs included in the in silico analysis. Available information about their association with male infertility as well as cancer is presented in brief for each of them. The red arrows indicate downregulation and upregulation of the lncRNAs as described in the figure. Figure made in Biorender.com.

Regarding the eight lncRNAs identified to be differentially expressed in many sub-types of male infertility (Figure 9)*, HOTAIR* (Hox transcript antisense intergenic RNA) is a well-studied lncRNA that plays an important role in tumor progression and development, as it is found to be overexpressed in many types of cancer [89], including breast cancer [90], lung cancer [91], hepatocellular carcinoma [92], etc. In male infertility, *HOTAIR* was found to be downregulated in patients with asthenozoospermia [35,41] and oligoasthenozoospermia [35]. Regarding its role, the downregulation of *HOTAIR* leads to both the downregulation of Nrf2 and the enhancement of oxidative stress that results in apoptosis and affects sperm motility and vitality. Thus, *HOTAIR* can be considered a protective lncRNA for spermatozoa, preventing them from oxidative stress and damage.

COX10 antisense RNA 1 (*COX10-AS1*) is another lncRNA that was found to be upregulated in patients with teratozoospermia [45] and asthenozoospermia [41], and is also deregulated in oligozoospermia [37]. Until now, little was known about the role of *COX10-AS1* in human diseases; however, many studies associate it with human cancers as well. A few studies have reported its upregulation in glioma [93,94] and osteosarcoma [95], and it is considered to act as an oncogene due to its complex interactions with miRNAs, inducing cell proliferation and inhibiting apoptosis [93,94].

RUSC1 Antisense RNA 1 is another lncRNA with a well-established role in tumorigenesis, which also acts as an oncogene. According to several studies, it has many mechanisms of action which lead to tumor growth. It promotes the proliferation of breast cancer cells by epigenetic silence of *KLF2* and *CDKN1A* [96], and is also involved in hepatocellular carcinoma by modulating Notch signaling through its interaction with miR-7-5p [97]. Its overexpression in cervical cancer tissues is associated with an increase in B-cell lymphoma 2 (Bcl-2 or *BCL2*) expression levels, by acting as a ceRNA of miR-744. It was also found to be upregulated in osteosarcoma in the same study as *COX10-AS1,* which is mentioned above [95]. There are many more studies exploring its interactions with miRNAs in tumor progression [97,98]. In the present systematic review, it was found to be upregulated in patients with Sertoli cell-only syndrome [29], and deregulated in asthenozoospermia [41] and oligozoospermia [37].

*SPATA42* or spermatogenesis associated 42 is a lncRNA which is highly expressed in the testis. It was found to be downregulated in two studies with NOA patients [27,29] and it is also deregulated in patients with asthenozoospermia [41], though no other publications are investigating its role or association with diseases. The same pattern is observed for lncRNA *LINC00710,* which is deregulated in NOA [27], oligozoospermia [37], and teratozoospermia [45]. This lncRNA is also highly expressed in the testis, but little information exists about its role or its involvement in specific cellular processes. There is only one study reporting its downregulation in lung adenocarcinoma [79].

*LINC01091* and *LINC01359* were also found to be deregulated in the three main subtypes of male infertility, namely asthenozoospermia [41], teratozoospermia [45], and oligozoospermia [37]. *LINC01091* also seems to have a role in cancer, as it regulates tumorigenesis and metastasis in gastric cancer [99], and is also implicated to have a role in prostate cancer [100]. Similarly, it has been reported that *LINC01359* could serve as a potential biomarker for hepatocellular carcinoma [101]. Finally, *LINC00905* is overexpressed in the testis and was found to be downregulated in patients with NOA [33], as well as differentially expressed in oligozoospermia [37] and asthenozoospermia [41]. It has been implicated to have a role in cervical cancer, leading to worse recurrence-free survival rates of patients [102].

Interestingly, many studies in the past have associated male infertility with an increased risk of many types of cancer. In a large study that included 76,083 infertile men, it was observed that infertile men had a much higher risk of developing testicular cancer and an increased risk of approximately 50% of developing a wide range of cancers, as compared to the control group [103]. Similarly, Eisenberg et al. (2013) [104] reported a 2.2-fold higher cancer risk for men with azoospermia, indicating common pathways involved in cancer and male infertility. Anderson et al. (2017) [105] also found the same results in a sample of 10,511 men. All of these studies suggest common molecular mechanisms and pathways, but no genetic cause or specific genes have yet been found that link cancer and male infertility. Thus, this systematic review and the in silico analysis performed provide preliminary data about lncRNAs and their target genes, which could be the missing link between cancer and male infertility. Many studies identified in the present review also showed that male infertility is caused by a disruption of cell proliferation and apoptosis mechanisms through lncRNA regulations. These two processes play a crucial role in cancer development and progression. In the case of male infertility, apoptosis can affect sperm production, as an imbalance between Sertoli and germ cells is observed [106]. In addition, apoptosis is essential for removing defective germ cells and ensuring high-quality sperm production [107]. Furthermore, spermatogenesis is a complex process that requires the proliferation of the spermatogonial sperm cells. Therefore, dysregulation of this process can lead to several problems, as indicated in many sub-types of infertility [108,109]. Finally, the p53 pathway was found to be an enriched term in genes targeted by the eight deregulated lncRNAs in male infertility. p53 is involved in meiotic recombination events, such as DNA double-strand break formation/repair, DNA recombination, cell cycle, etc. [109], and although p53 has been indicated to play a critical role in female fertility [110], its role in male infertility remains to be elucidated. Apart from the mechanisms reported here, others that have been proposed to be affected in both cancer and male infertility in the past include cell survival, cell fate, and genome maintenance [111], but further research is needed to clarify this point.

In addition to cancer, other pathways that were found to be affected by lncRNA regulation in men with infertility in the present systematic review were oxidative stress [35,37,38,40], ER stress [37], spermatogenesis and male gamete generation [31], and protein processing in the endoplasmic reticulum [31], as well as genes required for flagellum formation [30,36], coding for ion channels [43], etc.

### 4.3. Limitations

As with all systematic reviews, ours has several important limitations. First, as there is no official nomenclature for lncRNAs, it is possible that some information has been missed. Second, the studies included used normozoospermic men as controls based on the seminogram results, and not men proven to be fertile by previous pregnancy outcomes. Although this is a common approach used in most studies, it can lead to inconsistent results. Third, animal studies were excluded, as lncRNAs exhibit less sequence conservation across species in comparison with other RNAs [16], but some information may have been missed as well. Finally, since we excluded articles that were not published in English, we may have omitted relevant papers published in other languages.

Several limitations also arose from the existing studies included in this systematic review. One is that some studies, especially those using RNA sequencing or microarrays technology, do not present the full list of differentially expressed lncRNAs or do not provide information about their specific expression pattern (upregulation or downregulation in patients). Thus, the general terms “differentially regulated” or “deregulated” are used instead of “downregulated” and “upregulated” in this paper in cases when no specific information was available regarding the expression pattern of the lncRNAs by the original studies included. Although this is not informative and can lead to inconsistent results, even the information about the deregulation of lncRNAs, which is provided comprehensively here, can pave the road for future studies investigating the expression of these lncRNAs and their role in different sub-types of male infertility. Second, some of the studies included in this systematic review were performed on a rather small sample. This can lead to unreliable results, though we attempted to address this issue by selecting lncRNAs that were reported in multiple studies for the step of in silico analysis. Third, the papers included originated from just five countries, and more than half of them were studies performed in China, while some of the other countries were represented by only one study. It is noteworthy that there are no relevant data regarding the association of lncRNAs and male infertility in African populations. Therefore, the evidence is extremely limited in terms of geographical focus, which precludes us from performing comparisons regarding population genetics and ethnic differences, and indicating a gap for future research. Another limitation that arises from the studies included in the present systematic review is the fact that inadequate information is provided about the age and health status of the subjects in many studies. Most of them included men with a mean age of around 30 years, but some studies included much older individuals, or provided no information at all about age. Similarly, the health status of the individuals included (body mass index, alcohol consumption, other diseases, etc.) was not presented in sufficient detail in most studies. These problems can lead to nondefinitive conclusions, as, according to previous findings, an association is observed between male infertility and overall health status [112], indicating that other mechanisms and causes leading to male infertility are involved in these cases, and different lncRNAs may play a role. However, although this information was not provided in most studies, a large proportion of them excluded individuals with important underlying diseases, such as cancer, hormonal abnormalities, diabetes, etc. Thus, this problem may be addressed to some degree, but further investigation is required, as a few studies did not refer to their exclusion criteria at all. Finally, only three studies explored the impact of variants found in the lncRNA regions and their association with male infertility, also indicating a knowledge gap.

### 4.4. Strengths

Despite these limitations, our study has key strengths. To our knowledge, this is the first systematic review investigating the role of lncRNAs in male infertility, and also the first attempt to perform an in silico analysis including all the data that have been accumulated recently on the topic. We believe that this resource, as well as the list of lncRNAs found to be deregulated which is summarized here, will be of particular interest to the life science community, and will help biologists unravel the role of lncRNA in the pathogenesis of male infertility. Furthermore, it is the first study to shed light on the direct link between male infertility and carcinogenesis, providing candidate genes, lncRNAs, and their target genes, which could explain some of the observed shared genetic risks between these two conditions and have the great potential to be translated into clinical therapeutic or diagnostic approaches.

### 4.5. Directions for Future Research

Our systematic review points to several promising directions for future research. First, some lncRNAs were observed to be deregulated only on specific sub-types of male infertility; thus, their use as biomarkers to improve diagnosis and distinguish specific sub-types of male infertility from others should be investigated. Second, since there have not been many studies on different populations, more studies are required to clarify whether there are ethnic differences in deregulated lncRNAs in male infertility or in variants associated with male infertility. Third, some of the lncRNAs identified as deregulated in many subtypes of male infertility, such as *LINC00905,* are less studied, which implies that more research is needed. The biological functions of these lncRNAs remain to be explored. Fourth, most of the interactions between lncRNAs and miRNAs reported were predicted using bioinformatics models; thus, there is a need for further experimental studies investigating specific interactions and lncRNAs networks. Fifth, the limited number of studies exploring variants and mutations of lncRNAs regions indicate a knowledge gap, as no studies show whether these variants can have an impact on lncRNA expression or function through deregulating their interactions with other proteins or RNA molecules. Sixth, the identification of common deregulated pathways in cancer and male infertility is an important finding, and requires further investigation in order to discover the exact relationship between these two conditions. Furthermore, lncRNAs that have been associated with cancer in the past may provide a good candidate list for further investigation of their role in male infertility with functional experiments in the future. Finally, lncRNAs that were found to be perturbed only in one subtype of male infertility could be examined for their role in other sub-types, either by studying their expression levels or by further functional experiments, as some information maybe has been missed. Their role in other conditions associated with male infertility could also be investigated, such as varicocele-related male infertility, Klinefelter syndrome, Y chromosome microdeletions, etc.

We should especially highlight the need for studying the pathogenicity of male infertility caused by lncRNA variants. For many years, scientists mainly focused on variants found in protein-coding regions, in order to explore the molecular mechanisms involved in many diseases [113]. However, recently, it has been indicated that mutations in noncoding regions of the genome can also be involved in the pathogenesis of human diseases. Especially for lncRNAs, it has been proven that variants of regulatory regions, such as transcription binding sites, can affect the expression of lncRNAs, leading to the deregulation of the pathways mediated by the lncRNAs [114,115]. Furthermore, SNPs can affect the secondary structure of lncRNAs, impairing their interaction with miRNAs, RNA-binding proteins, etc. [113,116]. As in protein-coding genes, mutations in the lncRNAs region can disrupt the splicing process and affect the structure of lncRNAs, as well as their functionality [113]. Several studies have shown that variants of lncRNAs can lead to the development of several diseases through the mechanisms described above [113,116,117].

Therefore, future studies need to focus on the investigation of the role of lncRNAs variants on male infertility, using in vivo and in vitro approaches to identify the molecular mechanisms of their pathogenicity. At first, genome-wide association studies (GWAS), as well as whole-genome sequencing approaches, can be a valuable source for the identification of single nucleotide polymorphisms (SNPs) on lncRNAs associated with male infertility that can be verified for their role using experimental approaches [118]. Computational and bioinformatics tools can also be used to further prioritize these SNPs and to study their impact on lncRNAs’ structure, or to assess whether they affect interactions with other RNAs or proteins [78,119] that have been proven by previous studies to have a role in either the spermatogenesis process or other processes associated with fertilization. Then, the integration of GWAS and RNA sequencing data between large samples of normozoospermic and non-normozoospermic individuals can lead to the identification of variants affecting lncRNAs expression. It could also lead to the identification of variants acting as expression quantitative trait loci (eQTLs), thus having a greater possibility of contributing to the pathogenicity of male infertility through deregulation of specific pathways, such as meiosis, germline apoptosis, differentiation, etc. [120,121]. The expression of other genes previously identified to be targeted by lncRNAs could also be studied in order to explore the functional impact of variants and their role in male infertility. Cell lines are also a valuable tool for studying the effect of variants in combination with many different techniques, such as RNA pull-down and immunoprecipitation assays, to identify protein partners of the lncRNA of interest and interactions that may be disrupted by the presence of variants. Finally, in vivo studies on animal models carrying the potential disease-SNP allele can also be used to investigate the function of lncRNAs on male infertility, and especially to assess the impact of variants on the spermatogenesis process, fertility status, and testis function. However, it should be noted that this approach has several limitations, as most human lncRNAs are non-conserved [122]. All of the above approaches can be combined in order to obtain more robust information about specific variants of lncRNAs associated with male infertility, as well as their mechanisms of action. A long-term goal would also be to explore their utility in molecular diagnostics, or even therapeutics, by performing large-scale clinical trials.

Finally, the role of lncRNAs on other conditions associated with male infertility, apart from oligozoospermia, teratozoospermia, asthenozoospermia, and their combinations, is also a very interesting topic which requires further investigation. It has the potential to provide valuable information about the mechanisms leading to male infertility, as well as potential markers for distinguishing these conditions and successfully diagnosing them. As observed by this systematic review, lncRNAs contribute to varicocele-related male infertility, and for many of them, their expression levels are differentiated between normozoospermic and non-normozoospermic individuals [28,34,38,40]. Interestingly, varicocele-related lncRNAs, such as *GADD7* and *SLC7A11-AS1*, are associated with cell response to oxidative stress, indicating that this is a possible mechanism associated with the pathogenesis of varicocele and the presence of male infertility [34,40]. There is evidence for the role of lncRNAs on other conditions that were not examined in this systematic review, as well. More specifically, Klinefelter syndrome (KS) is a sex-chromosome disorder that leads to male infertility. LncRNAs play an important role in this case as well, as studies show that most RNAs that are differentially expressed in the testes of patients with KS and normozoospermic men are lncRNAs [123]. However, only a few individual lncRNAs have been detected as important players contributing to male infertility in Klinefelter syndrome, and their mechanism of action is unknown. For example, *GAS5* is overexpressed in KS patients, but it has been indicated in the past to be involved in several processes, such as cell proliferation, cell-to-cell signaling, inflammation, etc. [124]. Similarly, information about the role of lncRNAs in cases of male infertility attributed to specific causes, such as sperm DNA fragmentation, is extremely limited and scarce. Therefore, future studies should also focus on the study of lncRNAs regarding specific conditions associated with male infertility, apart from the classical conditions of asthenozoospermia, teratozoospermia, etc., as well as on its specific causes, such as sperm DNA fragmentation or Y chromosome microdeletions, in order to identify common patterns and potential biomarkers for diagnosis, as well as to shed light on the molecular mechanisms involved. All evidence indicates a regulatory role of lncRNAs on male infertility.

### 4.6. Challenges of Studying lncRNAs

It should be noted that, in general, the study of lncRNAs is challenging due to the fact that some of their characteristics and properties impose a significant complication for future studies exploring their role in spermatogenesis. More specifically, the greatest challenge for ascribing specific functions to lncRNAs and disentangling their role in male infertility is the low conservation of human lncRNAs [122]. Most of the human lncRNAs are non-conserved in other species; thus, it is difficult to obtain information about their function and their molecular mechanism of action based on evolutionary studies [125]. This problem can also lead to discrepancies and dissimilar results between in vitro studies and studies in animal models, as reported previously [115]. In some cases, as it is extremely difficult to find homologous sequences in other species for a lncRNA of interest, in vivo studies on animals cannot even be performed [126]. Second, most lncRNAs do not have large open reading frames that facilitate their identification or the prediction of their structure or functionality [127,128]. Many of these characteristics also affect the efficiency of the techniques that are generally used in molecular biology and genetics to study the functionality of RNAs, which could be used to explore their role in the spermatogenesis process. When a lncRNA is found to be deregulated in non-normozoospermic individuals, the next step usually involves loss-of-function studies to ascertain its functional role in male infertility. One popular approach nowadays is the CRISPR-Cas9 technology. CRISPR-Cas9 technology involves a guide RNA (gRNA) that targets the ORF, but, as lncRNAs have no ORF, modifications are required for using it [125]. In general, CRISPR-Cas9 technology is considered a demanding approach when used for lncRNAs, as it can cause deletion of other functional elements, leading to false results [126]. The RNAi (RNA interference) approach has also been proven to be less efficient for lncRNAs, potentially due to their localization or their low levels of expression. It is reported that in general, cycle threshold values are ≥35 for most lncRNAs. Therefore, the limited information available about the molecular mechanisms of lncRNAs involved in male infertility, as well as controversies in the field, can be attributed to the challenges required for their study. Advances are required in order to overcome these limitations and facilitate future studies on lncRNAs.

## 5. Conclusions

In conclusion, this is the first systematic review and in silico analysis to evaluate all published literature exploring the role of lncRNAs in male infertility. In this review, we attempted to provide a comprehensive summary of all lncRNAs which are deregulated in male infertility, or which play a role in it. In addition, this study indicates that eight lncRNAs may be dysregulated in many subtypes of male infertility. The in silico analysis performed also highlights the pathways regulated by these lncRNAs, as well as the molecular mechanisms involved in male infertility pathogenesis, showing common molecular mechanisms with cancer. Furthermore, as only 20 studies were included in this systematic review, it seems that the study of lncRNAs on male infertility is a new field, and there is much more to explore. It is hoped that this systematic review will be a basis for future research focusing on studying the role of specific lncRNAs, deciphering the molecular mechanisms associated with reduced fertility in men, and, perhaps, finding the missing link between male infertility and cancer.

## Figures and Tables

**Table 1:** Inclusion and exclusion criteria for the selection of articles included in the present systematic review.

Inclusion Criteria	Exclusion Criteria
Human participants	Animal and plant studies
Participants diagnosed with male infertility (seminogram)	Studies examining only the pregnancy outcome (no diagnosis of infertility)
Studies in English language	Reviews, perspectives, meta-analyses and other studies with non-original data
	Studies not providing sufficient details about data
	No appropriate control group for comparison between fertile and infertile men or mixed sample of fertile and infertile males
	Studies on other types of noncoding RNAs (circRNAs, piRNAs, etc.)
	Studies not in English language
	Studies about Klinefelter syndrome

**Table 2:** LncRNAs with a potential role in oligoasthenozoospermia, according to publications studying the expression profiles of lncRNAs and study characteristics.

LncRNAs	Reference	Methodology	Samples	Tissue	Change of Expression
*HOTAIR*	Zhang et al. (2015) [35]	qPCR	45 oligoasthenozoospermic patients, 45 healthy controls	Semen	Downregulated in patients

**Table 3:** LncRNAs with a potential role in teratoasthenozoospermia, according to publications studying the expression profiles of lncRNAs, and study characteristics.

LncRNAs	Reference	Methodology	Samples	Tissue	Change of Expression
*ANO1-AS2 (LINC02584)*	Saberiyan et al. (2020) [43]	qPCR	35 patients with TAZ, 34 people with normozoospermia (NZ, control)	Semen	Upregulated in patients
*CFAP44-AS1*	Kamel et al. (2022) [36]	qPCR	35 TAZ patients, 35 normozoospermic men	Semen	Downregulated in patients

**Table 4:** LncRNAs with a potential role in varicocele-related infertility, according to publications studying the expression profiles of lncRNAs, and study characteristics.

LncRNAs	Reference	Methodology	Samples	Tissue	Change of expression
*MIR210HG*	Ataabadi et al. (2020) [38]	RNA sequencing, qPCR	25 infertile men with varicocele, 17 fertile men as controls	Semen	Upregulation in patients
	Li et al. (2022) [28]	qPCR	188 VC patients, 92 healthy men	Seminal plasma	Upregulation in patients
*MLLT4-AS1*	Ataabadi et al. (2020) [38]	RNA sequencing, qPCR	25 infertile men with varicocele, 17 fertile men as controls	Semen	Upregulation in patients
*GADD7*	Zhao et al. (2017) [40]	qPCR	56 patients with varicocele, 28 healthy controls	Semen	Upregulated in patients
*SLC7A11-AS1 (isoform 6)*	Sanei-Ataabadi, Mowla and Nasr-Esfahani, (2020) [34]	qPCR	25 individuals with varicocele, 17 healthy donors with normal semen parameters	Semen	Upregulated in patients

**Table 5:** Experimentally validated lncRNAs–miRNAs interactions involved in male infertility.

Reference	Sub-Type of Male Infertility	lncRNA	miRNAs	Target Genes	Process Affected
Lü et al. (2015) [81]	NOA (MA, Hypo)	*NLC1-C*	miR-320a and miR-383	-	Spermatogenesis, cell apoptosis and proliferation
Su et al. (2019) [39]	NOA (Hypo)	*HOTTIP* (ceRNA)	miR-128-3p	*HOXA13*	Cell proliferation
Bo et al. (2020) [29]	NOA	*LINC00467*	miR-500-3p	*TDRD6, LRGUK*	Spermatogenesis (Male gamete generation)

**Table 6:** lncRNAs reported to be deregulated in two or more sub-types of male infertility.

LncRNAs	References	Sub-Types of Male Infertility	Regulation
*SPATA42*	Xie et al. (2020) [27]	NOA	Downregulated
	Bo et al. (2020) [29]	NOA	Downregulated
	Lu et al. (2020) [41]	Asthenozoospermia	-
*LINC00301*	Xie et al. (2020) [27]	NOA	Downregulated
	Sabetian et al. (2022) [33]	NOA	Downregulated
*ZNF503-AS1*	Xie et al. (2020) [27]	NOA	-
	Lu et al. (2020) [41]	Asthenozoospermia	-
*LINC00863*	Xie et al. (2020) [27]	NOA	-
	Lu et al. (2020) [41]	Asthenozoospermia	-
*PTOV1-AS2*	Xie et al. (2020) [27]	NOA	-
	Sun et al. (2021) [37]	Oligozoospermia	-
*LINC00710*	Xie et al. (2020) [27]	NOA	-
	Sun et al. (2021) [37]	Oligozoospermia	-
	Zhou and Wang, (2020) [45]	Teratozoospermia	-
*THUMPD3-AS1*	Xie et al. (2020) [27]	NOA	-
	Lu et al. (2020) [41]	Asthenozoospermia	-
*LINC00847*	Xie et al. (2020) [27]	NOA	-
	Sun et al. (2021) [37]	Oligozoospermia	-
*LINC00467*	Bo et al. (2020) [29]	NOA	Downregulated
	Zhou and Wang, (2020) [45]	Teratozoospermia	Downregulated
*LINC00173*	Bo et al. (2020) [29]	NOA	Upregulated
	Lu et al. (2020) [41]	Asthenozoospermia	-
*RUSC1-AS1*	Bo et al. (2020) [29]	NOA	Upregulated
	Lu et al. (2020) [41]	Asthenozoospermia	-
	Sun et al. (2021) [37]	Oligozoospermia	-
*FAM230B*	Sabetian et al. (2022) [33]	NOA	Downregulated
	Lu et al. (2020) [41]	Asthenozoospermia	-
*LINC00905*	Sabetian et al. (2022) [33]	NOA	Downregulated
	Lu et al. (2020) [41]	Asthenozoospermia	-
	Sun et al. (2021) [37]	Oligozoospermia	-
*MORC2-AS1*	Sabetian et al. (2022) [33]	NOA	Downregulated
	Sun et al. (2021) [37]	Oligozoospermia	-
*MIR210HG*	Ataabadi et al. (2020) [38]	Varicocele-related infertility	Upregulated
	Li et al. (2022) [28]	Varicocele-related infertility	Upregulated
*LINC01039*	Lu et al. (2020) [41]	Asthenozoospermia	-
	Sun et al. (2021) [37]	Oligozoospermia	-
*GLYCTK-AS1*	Lu et al. (2020) [41]	Asthenozoospermia	-
	Sun et al. (2021) [37]	Oligozoospermia	-
*COX10-AS1*	Lu et al. (2020) [41]	Asthenozoospermia	Upregulated
	Sun et al. (2021) [37]	Oligozoospermia	-
	Zhou and Wang (2020) [45]	Teratozoospermia	Upregulated
*LINC00894*	Lu et al. (2020) [41]	Asthenozoospermia	-
	Sun et al. (2021) [37]	Oligozoospermia	-
*TRIM52-AS1*	Lu et al. (2020) [41]	Asthenozoospermia	-
	Sun et al. (2021) [37]	Oligozoospermia	-
*HOTAIR*	Lu et al. (2020) [41]	Asthenozoospermia	-
	Zhang et al. (2015) [35]	Asthenozoospermia	Downregulated
	Zhang et al. (2015) [35]	Oligoasthenozoospermia	Downregulated
*CBR3-AS1*	Lu et al. (2020) [41]	Asthenozoospermia	-
	Sun et al. (2021) [37]	Oligozoospermia	-
*LINC01091*	Lu et al. (2020) [41]	Asthenozoospermia	-
	Sun et al. (2021) [37]	Oligozoospermia	-
	Zhou and Wang (2020) [45]	Teratozoospermia	-
*ZBED5-AS1*	Lu et al. (2020) [41]	Asthenozoospermia	-
	Sun et al. (2021) [37]	Oligozoospermia	-
*VIPR1-AS1*	Lu et al. (2020) [41]	Asthenozoospermia	-
	Sun et al. (2021) [37]	Oligozoospermia	-
*MYLK-AS1*	Lu et al. (2020) [41]	Asthenozoospermia	-
	Sun et al. (2021) [37]	Oligozoospermia	-
*FARSA-AS1*	Lu et al. (2020) [41]	Asthenozoospermia	-
	Sun et al. (2021) [37]	Oligozoospermia	-
*PPP3CB-AS1*	Lu et al. (2020) [41]	Asthenozoospermia	-
	Sun et al. (2021) [37]	Oligozoospermia	-
*LINC01270*	Lu et al. (2020) [41]	Asthenozoospermia	-
	Sun et al. (2021) [37]	Oligozoospermia	-
*SRRM2-AS1*	Lu et al. (2020) [41]	Asthenozoospermia	-
	Sun et al. (2021) [37]	Oligozoospermia	-
*LINC01359*	Lu et al. (2020) [41]	Asthenozoospermia	-
	Sun et al. (2021) [37]	Oligozoospermia	-
	Zhou and Wang (2020) [45]	Teratozoospermia	-
*UGDH-AS1*	Lu et al. (2020) [41]	Asthenozoospermia	-
	Sun et al. (2021) [37]	Oligozoospermia	-
*MIR4435-2HG*	Lu et al. (2020) [41]	Asthenozoospermia	-
	Sun et al. (2021) [37]	Oligozoospermia	-
*ANO1-AS2 (LINC02584)*	Saberiyan et al. (2020) [43]	Asthenozoospermia	Upregulated
	Saberiyan et al. (2020) [43]	Teratoasthenozoospermia	Upregulated
*CFAP44-AS1*	Kamel et al. (2022) [36]	Asthenozoospermia	Downregulated
	Kamel et al. (2022) [36]	Teratoasthenozoospermia	Downregulated
*RNF157-AS1*	Sun et al. (2021) [37]	Oligozoospermia	-
	Zhou and Wang (2020) [45]	Teratozoospermia	-

## Data Availability

Not applicable.

## Supplementary Materials

The following supporting information can be downloaded at: https://www.mdpi.com/article/10.3390/biology11101510/s1, Table S1: Characteristics of studies included in the present systematic review; Table S2: LncRNAs with a potential role in azoospermia according to publications studying the expression profiles of lncRNAs and study characteristics; Table S3: Differentially expressed lncRNAs based on results of specific studies and according to different subcategories of male infertility; Table S4: LncRNAs with a potential role in asthenozoospermia according to publications studying the expression profiles of lncRNAs and study characteristics; Table S5: LncRNAs with a potential role in teratozoospermia according to publications studying the expression profiles of lncRNAs and study characteristics; Table S6: LncRNAs with a potential role in oligozoospermia according to publications studying the expression profiles of lncRNAs; Table S7: Interactions between lncRNAs-miRNAs according to specific studies included in the present systematic review; Table S8: LncRNAs with a potential role in male infertility according to papers investigating genetic variants and study characteristics; Table S9: Genes targeted by the lncRNAs included in the in silico analysis.
