# Supplementary material for: The Role of Long Noncoding RNAs on Male Infertility: A Systematic Review and In Silico Analysis
Source: Biology (Basel). 2022 Oct 15;11(10):1510. doi: 10.3390/biology11101510 (PMC9598197; doi:10.3390/biology11101510)
Supplement: Supplementary file 1 [file biology-11-01510-s001.zip › Supplementary Table S2.pdf]

Supplementary Table S2. LncRNAs with a potential role in azoospermia according to publications studying the expression profiles of lncRNAs and study characteristics

LncRNAs	Referenc e	Methodolo gy	Samples	Tissue	Change of expression
NLC1-C (LINC00162)	Lü *et al.*, (2015) [26]	Microarray and qPCR	3 patients with MA, 2 with Hypo, 3 controls	Testis	Downregulat ed in NOA patients (MA, Hypo)
HOTTIP	Lü *et al.*, (2015) [26]	Microarray and qPCR	3 patients with MA, 2 with Hypo, 3 controls	Testis	Downregulat ed in NOA patients (MA, Hypo)
	Su *et al.*, (2019) [39]	qPCR	16 patients with Hypo, 16 control subjects	Testis	Downregulat ed in Hypo patients
FAM230B	Sabetian *et al.*, (2022) [33]	Microarray technology	27 NOA, 4 healthy individuals	Testis	Downregulat ed in patients
FER1L6-AS2	Sabetian *et al.*, (2022) [33]	Microarray technology	27 NOA, 4 healthy individuals	Testis	Downregulat ed in patients
FAM27C	Sabetian *et al.*, (2022) [33]	Microarray technology	27 NOA, 4 healthy individuals	Testis	Downregulat ed in patients
LINC00238	Sabetian *et al.*, (2022) [33]	Microarray technology	27 NOA, 4 healthy individuals	Testis	Downregulat ed in patients
LINC00301	Sabetian *et al.*, (2022) [33]	Microarray technology	27 NOA, 4 healthy individuals	Testis	Downregulat ed in patients
	Xie *et al.*, (2020) [27]	RNA sequencing and qPCR	RNA seq: 6 normozoosperm ic, 5 NOA patients (Sertoli cell-only syndrome); Validation: 96 NOA patients	Seminal plasma (extracellul ar vesicles)	Downregulat ed in patients
LINC00643	Sabetian *et al.*, (2022) [33]	Microarray technology	27 NOA, 4 healthy individuals	Testis	Downregulat ed in patients
LINC00654	Sabetian *et al.*, (2022) [33]	Microarray technology	27 NOA, 4 healthy individuals	Testis	Downregulat ed in patients
LINC00661	Sabetian *et al.*,	Microarray technology	27 NOA, 4 healthy individuals	Testis	Downregulat ed in patients

	(2022) [33]				
LINC00905	Sabetian *et al.*, (2022) [33]	Microarray technology	27 NOA, 4 healthy individuals	Testis	Downregulated in patients
MORC2-AS1	Sabetian *et al.*, (2022) [33]	Microarray technology	27 NOA, 4 healthy individuals	Testis	Downregulated in patients
LOC100505685	Xie *et al.*, (2020) [27]	RNA sequencing and qPCR	RNA seq: 6 normozoospermic, 5 NOA patients (Sertoli cell-only syndrome); Validation: 96 NOA patients	Seminal plasma (Extracellular vesicles)	Upregulated in patients
SPATA42	Xie *et al.*, (2020) [27]	RNA sequencing and qPCR	RNA seq: 6 normozoospermic, 5 NOA patients (Sertoli cell-only syndrome); Validation: 96 NOA patients	Seminal plasma (Extracellular vesicles)	Downregulated in patients
	Bo *et al.*, (2020) [29]	Microarray datasets and qPCR	3 sets: 30 NOA, 8 Normal (training); 31 NOA, 8 Normal (validation); 27 NOA, 4 Normal (validation)	Testis	Downregulated in patients
CCDC37-DT	Xie *et al.*, (2020) [27]	RNA sequencing and qPCR	RNA seq: 6 normozoospermic, 5 NOA patients (Sertoli cell-only syndrome); Validation: 96 NOA patients	Seminal plasma (Extracellular vesicles)	Downregulated in patients
GABRG3-AS1	Xie *et al.*, (2020) [27]	RNA sequencing and qPCR	RNA seq: 6 normozoospermic, 5 NOA patients (Sertoli cell-only syndrome); Validation: 96 NOA patients	Seminal plasma (Extracellular vesicles)	Downregulated in patients
LOC440934	Xie *et al.*, (2020) [27]	RNA sequencing and qPCR	RNA seq: 6 normozoospermic, 5 NOA patients (Sertoli cell-only syndrome);	Seminal plasma (Extracellular vesicles)	Downregulated in patients

			Validation: 96 NOA patients		
LOC101929088 (XR_927561.2)	Xie *et al.*, (2020) [27]	RNA sequencing and qPCR	RNA seq: 6 normozoospermic, 5 NOA patients (Sertoli cell-only syndrome); Validation: 96 NOA patients	Seminal plasma (Extracellular vesicles)	Upregulated in patients
LOC101929088 (XR_001745218.1)	Xie *et al.*, (2020) [27]	RNA sequencing and qPCR	RNA seq: 6 normozoospermic, 5 NOA patients (Sertoli cell-only syndrome); Validation: 96 NOA patients	Seminal plasma (Extracellular vesicles)	Upregulated in patients
LINC00343	Xie *et al.*, (2020) [27]	RNA sequencing and qPCR	RNA seq: 6 normozoospermic, 5 NOA patients (Sertoli cell-only syndrome); Validation: 96 NOA patients	Seminal plasma (Extracellular vesicles)	Downregulated in patients
LINC00467	Bo *et al.*, (2020) [29]	Microarray datasets and qPCR	3 sets: 30 NOA, 8 Normal (training); 31 NOA, 8 Normal (validation); 27 NOA, 4 Normal (validation)	Testis	Downregulated in patients
LINC00884	Bo *et al.*, (2020) [29]	Microarray datasets and qPCR	3 sets: 30 NOA, 8 Normal (training); 31 NOA, 8 Normal (validation); 27 NOA, 4 Normal (validation)	Testis	Upregulated in patients
LEMD1-AS1	Bo *et al.*, (2020) [29]	Microarray datasets and qPCR	3 sets: 30 NOA, 8 Normal (training); 31 NOA, 8 Normal (validation); 27 NOA, 4 Normal (validation)	Testis	Upregulated in patients
ZFAS1	Bo *et al.*, (2020) [29]	Microarray datasets and qPCR	3 sets: 30 NOA, 8 Normal (training); 31 NOA, 8 Normal (validation); 27 NOA, 4 Normal (validation)	Testis	Upregulated in patients

CSNK1G2-AS1	Bo *et al.*, (2020) [29]	Microarray datasets and qPCR	3 sets: 30 NOA, 8 Normal (training); 31 NOA, 8 Normal (validation); 27 NOA, 4 Normal (validation)	Testis	Downregulated in patients
ZNF295-AS1	Bo *et al.*, (2020) [29]	Microarray datasets and qPCR	3 sets: Sertoli cell-only, 8 normal (training); Sertoli cell-only, 8 Normal (validation), Sertoli cell-only, 4 Normal (validation)	Testis	Downregulated in patients
DSCR8	Bo *et al.*, (2020) [29]	Microarray datasets and qPCR	3 sets: Sertoli cell-only, 8 normal (training); Sertoli cell-only, 8 Normal (validation), Sertoli cell-only, 4 Normal (validation)	Testis	Downregulated in patients
LINC00173	Bo *et al.*, (2020) [29]	Microarray datasets and qPCR	3 sets: Sertoli cell-only, 8 normal (training); Sertoli cell-only, 8 Normal (validation), Sertoli cell-only, 4 Normal (validation)	Testis	Upregulated in patients
RUSC1-AS1	Bo *et al.*, (2020) [29]	Microarray datasets and qPCR	3 sets: Sertoli cell-only, 8 normal (training); Sertoli cell-only, 8 Normal (validation), Sertoli cell-only, 4 Normal (validation)	Testis	Upregulated in patients
