# Supplementary material for: The Role of Long Noncoding RNAs on Male Infertility: A Systematic Review and In Silico Analysis
Source: Biology (Basel). 2022 Oct 15;11(10):1510. doi: 10.3390/biology11101510 (PMC9598197; doi:10.3390/biology11101510)
Supplement: Supplementary file 1 [file biology-11-01510-s001.zip › Supplementary Table S4.pdf]

Supplementary Table S4. LncRNAs with a potential role in asthenozoospermia according to publications studying the expression profiles of lncRNAs and study characteristics

LncRNAs	Reference	Methodology	Samples	Tissue	Change of expression
HOTAIR	Zhang *et al.*, (2015) [35]	qPCR	45 asthenozoospermic patients, 45 healthy controls	Semen	Downregulated in patients
LNC32058	Zhang *et al.*, (2019) [31]	RNA sequencing and qPCR	36 asthenozoospermic males, 48 healthy males	Semen	Upregulated in patients
LNC09522	Zhang *et al.*, (2019) [31]	RNA sequencing and qPCR	36 asthenozoospermic males, 48 healthy males	Semen	Upregulated in patients
LNC98487	Zhang *et al.*, (2019) [31]	RNA sequencing and qPCR	36 asthenozoospermic males, 48 healthy males	Semen	Upregulated in patients
ANO1-AS2 (LINC02584)	Saberiyan *et al.*, (2020) [43]	qPCR	32 patients with AZ, 34 people with normozoospermia (NZ, control)	Semen	Upregulated in patients
IGF2-AS	Lu *et al.*, (2020) [41]	RNA sequencing and qPCR	25 men with asthenozoospermia, 25 male healthy individuals	Seminal plasma (exosomes)	Downregulated in patients
LINC00893	Lu *et al.*, (2020) [41]	RNA sequencing and qPCR	25 men with asthenozoospermia, 25 male healthy individuals	Seminal plasma (exosomes)	Downregulated in patients
LINC00667	Lu *et al.*, (2020) [41]	RNA sequencing and qPCR	25 men with asthenozoospermia, 25 male healthy individuals	Seminal plasma (exosomes)	Upregulated in patients
MIR497HG	Lu *et al.*, (2020) [41]	RNA sequencing and qPCR	25 men with asthenozoospermia, 25 male healthy individuals	Seminal plasma (exosomes)	Downregulated in patients
COX10-AS1	Lu *et al.*, (2020) [41]	RNA sequencing and qPCR	25 men with asthenozoospermia, 25 male healthy individuals	Seminal plasma (exosomes)	Upregulated in patients
AC010247.2	Lu *et al.*, (2020) [41]	RNA sequencing and qPCR	25 men with asthenozoospermia, 25 male	Seminal plasma	Downregulated in patients

			healthy individuals	(exosomes)	
AC009965.1	Lu *et al.*, (2020) [41]	RNA sequencing and qPCR	25 men with asthenozoospermia, 25 male healthy individuals	Seminal plasma (exosomes)	Upregulated in patients
PCED1B-AS1	Lu *et al.*, (2020) [41]	RNA sequencing and qPCR	25 men with asthenozoospermia, 25 male healthy individuals	Seminal plasma (exosomes)	Upregulated in patients
AL358234.1	Lu *et al.*, (2020) [41]	RNA sequencing and qPCR	25 men with asthenozoospermia, 25 male healthy individuals	Seminal plasma (exosomes)	Upregulated in patients
AC121764.1	Lu *et al.*, (2020) [41]	RNA sequencing and qPCR	25 men with asthenozoospermia, 25 male healthy individuals	Seminal plasma (exosomes)	Upregulated in patients
AC019117.2	Lu *et al.*, (2020) [41]	RNA sequencing and qPCR	25 men with asthenozoospermia, 25 male healthy individuals	Seminal plasma (exosomes)	Upregulated in patients
AC112176.1	Lu *et al.*, (2020) [41]	RNA sequencing and qPCR	25 men with asthenozoospermia, 25 male healthy individuals	Seminal plasma (exosomes)	Upregulated in patients
AC022398.1	Lu *et al.*, (2020) [41]	RNA sequencing and qPCR	25 men with asthenozoospermia, 25 male healthy individuals	Seminal plasma (exosomes)	Upregulated in patients
LSAMP-AS1	Lu *et al.*, (2020) [41]	RNA sequencing and qPCR	25 men with asthenozoospermia, 25 male healthy individuals	Seminal plasma (exosomes)	Upregulated in patients
AC025300.1	Lu *et al.*, (2020) [41]	RNA sequencing and qPCR	25 men with asthenozoospermia, 25 male healthy individuals	Seminal plasma (exosomes)	Upregulated in patients
LINC02042	Lu *et al.*, (2020) [41]	RNA sequencing and qPCR	25 men with asthenozoospermia, 25 male healthy individuals	Seminal plasma (exosomes)	Upregulated in patients
AC011444.3	Lu *et al.*, (2020) [41]	RNA sequencing and qPCR	25 men with asthenozoospermia, 25 male	Seminal plasma (exosomes)	Downregulated in patients

			healthy individuals		
AC019193.3	Lu *et al.*, (2020) [41]	RNA sequencing and qPCR	25 men with asthenozoospermia, 25 male healthy individuals	Seminal plasma (exosomes)	Downregulated in patients
AC068389.1	Lu *et al.*, (2020) [41]	RNA sequencing and qPCR	25 men with asthenozoospermia, 25 male healthy individuals	Seminal plasma (exosomes)	Downregulated in patients
AL359771.1	Lu *et al.*, (2020) [41]	RNA sequencing and qPCR	25 men with asthenozoospermia, 25 male healthy individuals	Seminal plasma (exosomes)	Downregulated in patients
AC008735.2	Lu *et al.*, (2020) [41]	RNA sequencing and qPCR	25 men with asthenozoospermia, 25 male healthy individuals	Seminal plasma (exosomes)	Downregulated in patients
AC009093.4	Lu *et al.*, (2020) [41]	RNA sequencing and qPCR	25 men with asthenozoospermia, 25 male healthy individuals	Seminal plasma (exosomes)	Downregulated in patients
LINC02320	Lu *et al.*, (2020) [41]	RNA sequencing and qPCR	25 men with asthenozoospermia, 25 male healthy individuals	Seminal plasma (exosomes)	Downregulated in patients
AC022098.1	Lu *et al.*, (2020) [41]	RNA sequencing and qPCR	25 men with asthenozoospermia, 25 male healthy individuals	Seminal plasma (exosomes)	Downregulated in patients
AC022098.2	Lu *et al.*, (2020) [41]	RNA sequencing and qPCR	25 men with asthenozoospermia, 25 male healthy individuals	Seminal plasma (exosomes)	Downregulated in patients
AL513320.1	Lu *et al.*, (2020) [41]	RNA sequencing and qPCR	25 men with asthenozoospermia, 25 male healthy individuals	Seminal plasma (exosomes)	Downregulated in patients
LINC00574	Saberiyan *et al.*, (2021) [30]	qPCR	31 AZ patients, 32 normozoospermic controls (NZ)	Semen	Upregulated in patients
CFAP44-AS1	Kamel *et al.*, (2022) [36]	qPCR	34 AZ patients, 35 normozoospermic men	Semen	Downregulated in patients
