# Supplementary material for: The Role of Long Noncoding RNAs on Male Infertility: A Systematic Review and In Silico Analysis
Source: Biology (Basel). 2022 Oct 15;11(10):1510. doi: 10.3390/biology11101510 (PMC9598197; doi:10.3390/biology11101510)
Supplement: Supplementary file 1 [file biology-11-01510-s001.zip › Supplementary Table S5.pdf]

Supplementary Table S5. LncRNAs with a potential role in teratozoospermia according to publications studying the expression profiles of lncRNAs and study characteristics

LncRNAs	Reference	Methodology	Samples	Tissue	Change of expression
LINC00266-1	Zhou and Wang, (2020) [45]	Gene expression array	8 teratozoospermic, 13 normozoospermic	Semen	Upregulated in patients
LINC00467	Zhou and Wang, (2020) [45]	Gene expression array	8 teratozoospermic, 13 normozoospermic	Semen	Downregulated in patients
ADARB2-AS1	Zhou and Wang, (2020) [45]	Gene expression array	8 teratozoospermic, 13 normozoospermic	Semen	Upregulated in patients
AP000525	Zhou and Wang, (2020) [45]	Gene expression array	8 teratozoospermic, 13 normozoospermic	Semen	Upregulated in patients
AC008088	Zhou and Wang, (2020) [45]	Gene expression array	8 teratozoospermic, 13 normozoospermic	Semen	Downregulated in patients
COX10-AS1	Zhou and Wang, (2020) [45]	Gene expression array	8 teratozoospermic, 13 normozoospermic	Semen	Upregulated in patients
HLA-F-AS1	Zhou and Wang, (2020) [45]	Gene expression array	8 teratozoospermic, 13 normozoospermic	Semen	Downregulated in patients
LINC00466	Zhou and Wang, (2020) [45]	Gene expression array	8 teratozoospermic, 13 normozoospermic	Semen	Upregulated in patients
EPB41L4A-AS1	Zhou and Wang, (2020) [45]	Gene expression array	8 teratozoospermic, 13 normozoospermic	Semen	Downregulated in patients
LINC00347	Zhou and Wang, (2020) [45]	Gene expression array	8 teratozoospermic, 13 normozoospermic	Semen	Downregulated in patients
LINC00276	Zhou and Wang, (2020) [45]	Gene expression array	8 teratozoospermic, 13 normozoospermic	Semen	Upregulated in patients
AL022344	Zhou and Wang, (2020) [45]	Gene expression array	8 teratozoospermic, 13 normozoospermic	Semen	Upregulated in patients
C12orf77	Zhou and Wang,	Gene expression array	8 teratozoospermic,	Semen	Upregulated in patients

	(2020) [45]		13 normozoospermic		
ST7-AS2	Zhou and Wang, (2020) [45]	Gene expression array	8 teratozoospermic, 13 normozoospermic	Semen	Downregulated in patients
PRICKLE2-AS3	Zhou and Wang, (2020) [45]	Gene expression array	8 teratozoospermic, 13 normozoospermic	Semen	Upregulated in patients
ZBTB20-AS1	Zhou and Wang, (2020) [45]	Gene expression array	8 teratozoospermic, 13 normozoospermic	Semen	Downregulated in patients
IGSF11-AS1	Zhou and Wang, (2020) [45]	Gene expression array	8 teratozoospermic, 13 normozoospermic	Semen	Downregulated in patients
AC092620	Zhou and Wang, (2020) [45]	Gene expression array	8 teratozoospermic, 13 normozoospermic	Semen	Upregulated in patients
PARD6G-AS1	Zhou and Wang, (2020) [45]	Gene expression array	8 teratozoospermic, 13 normozoospermic	Semen	Downregulated in patients
MALAT1	Zhou and Wang, (2020) [45]	Gene expression array	8 teratozoospermic, 13 normozoospermic	Semen	Downregulated in patients
SLIT2-IT1	Zhou and Wang, (2020) [45]	Gene expression array	8 teratozoospermic, 13 normozoospermic	Semen	Downregulated in patients
FAM13A-AS1	Zhou and Wang, (2020) [45]	Gene expression array	8 teratozoospermic, 13 normozoospermic	Semen	Upregulated in patients
ARHGEF26-AS1	Zhou and Wang, (2020) [45]	Gene expression array	8 teratozoospermic, 13 normozoospermic	Semen	Upregulated in patients
FAM181A-AS1	Zhou and Wang, (2020) [45]	Gene expression array	8 teratozoospermic, 13 normozoospermic	Semen	Downregulated in patients
LZTS1-AS1	Zhou and Wang, (2020) [45]	Gene expression array	8 teratozoospermic, 13 normozoospermic	Semen	Upregulated in patients
