# Supplementary material for: The Role of Long Noncoding RNAs on Male Infertility: A Systematic Review and In Silico Analysis
Source: Biology (Basel). 2022 Oct 15;11(10):1510. doi: 10.3390/biology11101510 (PMC9598197; doi:10.3390/biology11101510)
Supplement: Supplementary file 1 [file biology-11-01510-s001.zip › Supplementary Table S6.pdf]

Supplementary Table S6. LncRNAs with a potential role in oligozoospermia according to publications studying the expression profiles of lncRNAs

LncRNAs	Reference	Methodology	Samples	Tissue	Change of expression
lnc-GNS-3	Sun *et al.*, (2021) [37]	RNA sequencing, qPCR	6 oligozoospermic, 6 normozoospermic	Semen	Upregulated in patients
XLOC_515910	Sun *et al.*, (2021) [37]	RNA sequencing, qPCR	6 oligozoospermic, 6 normozoospermic	Semen	Upregulated in patients
lnc-SERHL2-7	Sun *et al.*, (2021) [37]	RNA sequencing, qPCR	6 oligozoospermic, 6 normozoospermic	Semen	Upregulated in patients
lnc-IL31RA-1	Sun *et al.*, (2021) [37]	RNA sequencing, qPCR	6 oligozoospermic, 6 normozoospermic	Semen	Upregulated in patients
lnc-NLRP2-2	Sun *et al.*, (2021) [37]	RNA sequencing, qPCR	6 oligozoospermic, 6 normozoospermic	Semen	Upregulated in patients
lnc-CDK12-2	Sun *et al.*, (2021) [37]	RNA sequencing, qPCR	6 oligozoospermic, 6 normozoospermic	Semen	Upregulated in patients
XLOC_1093926	Sun *et al.*, (2021) [37]	RNA sequencing, qPCR	6 oligozoospermic, 6 normozoospermic	Semen	Upregulated in patients
lnc-LRRC38-2	Sun *et al.*, (2021) [37]	RNA sequencing, qPCR	6 oligozoospermic, 6 normozoospermic	Semen	Upregulated in patients
lnc-FOXN1-1	Sun *et al.*, (2021) [37]	RNA sequencing, qPCR	6 oligozoospermic, 6 normozoospermic	Semen	Upregulated in patients
lnc-SLC46A2-1	Sun *et al.*, (2021) [37]	RNA sequencing, qPCR	6 oligozoospermic, 6 normozoospermic	Semen	Upregulated in patients
lnc-CSNK1A1-7	Sun *et al.*, (2021) [37]	RNA sequencing, qPCR	6 oligozoospermic, 6 normozoospermic	Semen	Upregulated in patients
lnc-PHLDB1-1	Sun *et al.*, (2021) [37]	RNA sequencing, qPCR	6 oligozoospermic, 6 normozoospermic	Semen	Upregulated in patients

lnc-KB-1980E6.3.1-6	Sun *et al.*, (2021) [37]	RNA sequencing, qPCR	6 oligozoospermic, 6 normozoospermic	Semen	Upregulated in patients
lnc-LAT2-2	Sun *et al.*, (2021) [37]	RNA sequencing, qPCR	6 oligozoospermic, 6 normozoospermic	Semen	Upregulated in patients
XLOC_2394941	Sun *et al.*, (2021) [37]	RNA sequencing, qPCR	6 oligozoospermic, 6 normozoospermic	Semen	Upregulated in patients
lnc-EEF1B2-3	Sun *et al.*, (2021) [37]	RNA sequencing, qPCR	6 oligozoospermic, 6 normozoospermic	Semen	Upregulated in patients
lnc-AP3S1-15	Sun *et al.*, (2021) [37]	RNA sequencing, qPCR	6 oligozoospermic, 6 normozoospermic	Semen	Upregulated in patients
lnc-SLA2-1	Sun *et al.*, (2021) [37]	RNA sequencing, qPCR	6 oligozoospermic, 6 normozoospermic	Semen	Upregulated in patients
lnc-RAPH1-6	Sun *et al.*, (2021) [37]	RNA sequencing, qPCR	6 oligozoospermic, 6 normozoospermic	Semen	Upregulated in patients
lnc-TICAM1-1	Sun *et al.*, (2021) [37]	RNA sequencing, qPCR	6 oligozoospermic, 6 normozoospermic	Semen	Upregulated in patients
ITGA9-AS1	Sun *et al.*, (2021) [37]	RNA sequencing, qPCR	6 oligozoospermic, 6 normozoospermic	Semen	Downregulated in patients
