# Supplementary material for: The Role of Long Noncoding RNAs on Male Infertility: A Systematic Review and In Silico Analysis
Source: Biology (Basel). 2022 Oct 15;11(10):1510. doi: 10.3390/biology11101510 (PMC9598197; doi:10.3390/biology11101510)
Supplement: Supplementary file 1 [file biology-11-01510-s001.zip › Supplementary Table S8.pdf]

Supplementary Table S8: LncRNAs with a potential role in male infertility according to papers investigating genetic variants and study characteristics

LncRNAs	Reference	Methodology	Samples	Tissue
LOC100507205	Eggers *et al.*, (2015) [32]	Microarray technology, MLPA Analysis	19 men with histologically confirmed meiotic arrest, healthy individuals (information from database)	Peripheral leucocytes (Blood)
LINC02231	Kyrgiafini *et al.*, (2020) [42]	SNP screening array (genotyping), Bioinformatics	83 non-normozoospermic (oligozoospermic, asthenozoospermic, teratozoospermic or combinations), 76 normozoospermic individuals	Semen
LINC00347	Kyrgiafini *et al.*, (2020) [42]	SNP screening array (genotyping), Bioinformatics	83 non-normozoospermic (oligozoospermic, asthenozoospermic, teratozoospermic or combinations), 76 normozoospermic individuals	Semen
LINC02134	Kyrgiafini *et al.*, (2020) [42]	SNP screening array (genotyping), Bioinformatics	83 non-normozoospermic (oligozoospermic, asthenozoospermic, teratozoospermic or combinations), 76 normozoospermic individuals	Semen
NCRNA00157/CHODL-AS1	Kyrgiafini et al., (2020) [42]	SNP screening array (genotyping), Bioinformatics	83 non-normozoospermic (oligozoospermic, asthenozoospermic, teratozoospermic or combinations), 76 normozoospermic individuals	Semen
LINC02493	Kyrgiafini *et al.*, (2020) [42]	SNP screening array (genotyping), Bioinformatics	83 non-normozoospermic (oligozoospermic, asthenozoospermic, teratozoospermic or combinations), 76 normozoospermic individuals	Semen

Lnc-CASK-1	Kyrgiafini *et al.*, (2020) [42]	SNP screening array (genotyping), Bioinformatics	83 non-normozoospermic (oligozoospermic, asthenozoospermic, teratozoospermic or combinations), 76 normozoospermic individuals	Semen
LINC01641	Cerván-Martín *et al.*, (2021) [44]	Genotyping (TaqMan allelic discrimination technology), Bioinformatics analysis	674 infertile men with azoospermia or severe oligospermia (480 NOA and 194 SO men), 700 men representative of the general population (with self- reported fatherhood) and 358 men with proven normal sperm concentration	Peripheral blood
RP11- 38C6.2	Cerván-Martín *et al.*, (2021) [44]	Genotyping (TaqMan allelic discrimination technology), Bioinformatics analysis	674 infertile men with azoospermia or severe oligospermia (480 NOA and 194 SO men), 700 men representative of the general population (with self- reported fatherhood) and 358 men with proven normal sperm concentration	Peripheral blood
